# First evidence of octacalcium phosphate@osteocalcin nanocomplex as skeletal bone component directing collagen triple–helix nanofibril mineralization

**DOI:** 10.1038/s41598-018-31983-5

**Published:** 2018-09-12

**Authors:** Paul Simon, Daniel Grüner, Hartmut Worch, Wolfgang Pompe, Hannes Lichte, Thaqif El Khassawna, Christian Heiss, Sabine Wenisch, Rüdiger Kniep

**Affiliations:** 1 Max-Planck-Institut für Chemische Physik fester Stoffe, Nöthnitzer Str. 40, 01187 Dresden, Germany; 2 Forschungszentrum Jülich GmbH, Institute of Energy and Climate Research, IEK-2, 52425 Jülich, Germany; 3 Institute of Materials Science, Technical University of Dresden, Helmholtzstr. 7, 01069 Dresden, Germany; 4 Institute of Structure Physics, Technical University of Dresden, Zum Triebenberg 50, 01328 Dresden Zaschendorf, Germany; 5 Experimental Trauma Surgery, Faculty of Medicine, Justus-Liebig University of Giessen, Aulweg 128, Giessen, 35392 Germany; 6 Department of Trauma, Hand and Reconstructive Surgery, University Hospital of Giessen-Marburg, Giessen, Germany; 7 Clinic of Small animals, c/o Institute of Veterinary Anatomy, Justus-Liebig University of Giessen, Giessen, Germany

## Abstract

Tibia trabeculae and vertebrae of rats as well as human femur were investigated by high-resolution TEM at the atomic scale in order to reveal snapshots of the morphogenetic processes of local bone ultrastructure formation. By taking into account reflections of hydroxyapatite for Fourier filtering the appearance of individual alpha–chains within the triple–helix clearly shows that bone bears the feature of an intergrowth composite structure extending from the atomic to the nanoscale, thus representing a molecular composite of collagen and apatite. Careful Fourier analysis reveals that the non–collagenous protein osteocalcin is present directly combined with octacalcium phosphate. Besides single spherical specimen of about 2 nm in diameter, osteocalcin is spread between and over collagen fibrils and is often observed as pearl necklace strings. In high-resolution TEM, the three binding sites of the γ-carboxylated glutamic acid groups of the mineralized osteocalcin were successfully imaged, which provide the chemical binding to octacalcium phosphate. Osteocalcin is attached to the collagen structure and interacts with the Ca–sites on the (100) dominated hydroxyapatite platelets with Ca-Ca distances of about 9.5 Å. Thus, osteocalcin takes on the functions of Ca–ion transport and suppression of hydroxyapatite expansion.

## Introduction

Since the first report on bone structure by Havers^1^ the structural hierarchy described for bone comprised mostly five levels and over the years became more refined and was extended to seven^2^ and nowadays to even nine levels^3^. The basic structural unit of type I collagen, a triple–helix consisting of two identical *alpha*_1_ –chains and an *alpha*_2_–chain, is an insoluble molecule with about 300 nm in length and 1.5 nm in thickness^4^. Type I collagen is an amphiphilic molecule. In the presence of divalent electrolytes (Ca^2+^), it is positively charged near neutral pH due to the excess of basic residues in the amino acids sequence. As shown by Freudenberg *et al*.^5^, the isoelectric point shifts with increasing ionic strength in CaCl_2_ solutions from pH 7.5 to above pH 9. Bone consists of mineralized collagen containing about 65 wt.% calcium phosphate, 25 wt.% organic molecules, mainly type I collagen and 10 wt.%water^2,6,7^.

Non-collagenous proteins (NCPs) amount to about 10–15 wt.% of the organic part^8,9^. NCPs can be divided into five groups. About 25% of the NCPs are calcium binding serum proteins such as albumin with a molecular weight of 69 kilo Dalton (kD), and fetuin precursor α2-HS glycoprotein 2q11-13. Thus, this group encompasses only two different proteins. Proteoglycans (13 proteins) with molecular weights ranging from 59 up to several million kD, and glycosated proteins (7 proteins) with molecular weights from 21 up to 1920 kD are the largest group in number. The fourth group belongs to the cell binding proteins (9 members) from 44 to 450 kD. Bone sialoprotein (46–75 kD) from this group is a significant component of the bone extracellular matrix and constitutes approximately 8% of all non-collagenous proteins found in bone and cementum^7^. Another prominent representative of this group is osteopontin (44–75 kD). Both anchor osteoclasts and show very strong Ca^2+^ binding. The fifth group belongs to the dicarboxylic glutamyl or Gla proteins with acidic carboxylic groups (4 members/proteins) with molecular weights from 5–72 kD with osteocalcin (OC) being the most important member of this family.

Besides type I collagen (85–90 wt.%), OC is the next most abundant protein within the organic matrix of mature human bone and plays a major role as structure directing molecule^10,11^ and in the regulation of osteoblast and osteoclast activity^12^. The content of osteocalcin ranges from 0.028 (human) to 0.2–0.25 wt.% (bovine) for dry bone. Most species cluster in the 0.14–0.19 wt.% range^11^. OC (molecular weight 5.2–5.9 kD) acts as a calcium binding protein, which is largely unstructured in aqueous solution, but transforms to a folded state with an almost globular structure with a diameter of about 1.7–2.0 nm in the presence of physiological concentrations of calcium^13^. OC is present at the surface of mineralized collagen fibrils. Investigations revealed the association of osteocalcin with type I collagen where the binding proved to be reversible^14,15^. Inside the collagen fibrils osteocalcin has been detected in part of the gap region and the neighboring overlap zone. Thus, we assume that OC mediates nucleation and growth of platelet–shaped hydroxyapatite (HAP) crystals^16^. The crystal structure of the porcine OC–Ca^2+^ complex reveals a negatively charged protein surface that coordinates up to five calcium ions *via* three Gla– and Asp–residues (Gla = γ–carboxylated glutamic acid from helix α_1_, Asp = aspartic acid from helix α_2_). The best fit for a monomer molecule attached to HAP is a plane running parallel to the *c–*axis^17^. Osteocalcin participates in regulation of mineralization since observations on the osteocalcin knockout mouse imply that osteocalcin may function to restrict bone formation. Molecular dynamics simulations reveal that the best correlation of the distance between the uncoordinated Gla–oxygen atoms (COOH groups) of OC is the inter calcium distance of 9.43 Å in the hydroxyapatite crystal structure (rather than 5.44 Å as previously suggested by *Hauschka*)^18^. The increased availability of bonding sites (9.43 Å // parallel rows) on the (100) plane of HAP may explain the preferential binding of osteocalcin on this face^12^.

The role of octacalcium phosphate (OCP) in bone formation is hotly debated^19–21^. OCP is considered as the water–rich precursor phase of HAP prevalent at acidic conditions where OCP nuclei may initiate the HAP mineralization by offering epitaxic interfaces^22–25^. However, OCP is detected only during pathological calcification where the pH is often relatively low. Therefore, the involvement of other precursors was suggested or the formation of an initial amorphous calcium phosphate phase followed by transformation to HAP^26,27^. In addition, even the presence of semicrystalline HAP nanoplatelets was observed at the mineralization front of bone^28^.

Our intention was to reveal the nanostructural relationships between the collagen fibrillar structure and osteocalcin as well as the nucleation and deposition mechanisms for octacalcium phosphate and HAP growth. In this work, we used the combination of focused ion beam (FIB) cuts and TEM. We also added results of diamond cut samples (ultramicrotomy) in order to rule out artefacts, which may be evoked by FIB cuts. The present high–resolution TEM (HR–TEM) investigation was performed in order to contribute to open questions concerning the interplay between collagen, HAP, OC and OCP. Because of the complex structure of bone, characterized by various levels of hierarchy, the investigations were restricted to the basic composite units on the fibril level from the atomic to the nanoscale.

In the following, we present results of three different bone samples. First of all a FIB cut of rat tibia where the structures to be revealed are emphasized as we think due to the sandblasting effect of the gallium ions. Furthermore, in order to ensure that the observed structures are not artefacts produced by the FIB method we also checked conventional diamond ultramicrotomy cuts of rat vertebra. Because of the fundamental significance of the work, and as additional prove for the correctness of our findings, we carried out also experiments on human bone. With the investigation of three different samples prepared by two different techniques (FIB and ultramicrotomy) on two different species (rat and human) and three different locations (femur, vertebra, tibia) we hope to provide a solid base for our results. At least, we carried out also *in vitro* experiments with osteocalcin and OCP to prove that the observed structures are not artefacts or stemming from other non-collagenous proteins but definitely exclusively from osteocalcin.

## Materials and Methods

### Animal model

Eighteen female Sprague–Dawley rats (10 weeks old, 250–290 g body–weight) were purchased from Charles River (Sulzfeld, Germany). The rats were maintained under standard laboratory conditions and underwent an acclimatization period of 4 weeks before the experimental procedures. Animals housing was at 22 ± 2 °C with relative humidity of 45–65% under a 12 h light/dark cycle and a free access to food and tap water. The rats were housed until mature age (M = months: 16 M). Presurgical anesthesia consisted of intravenous injection 103 of 62.5 mg ketamine (Hostaket®, Hoechst, Germany) and 7.5 mg xylazine (Rompun®, Bayer, Germany) *per* kg body weight. Painkillers were delivered subcutaneously before surgery and up to five days post-surgery using Metacam® (Meloxicam, Boehringer Ingelheim Pharma GmbH, Germany) with 0.2 mg/kg dosage. Daily monitoring revealed no need for further painkillers or antibiotics post operatively^29^.

### Ultramicrotomy of rat vertebra

For ultrastructural examinations, small trabecular samples from were post-fixed at 21 °C for 24 h in Yellow Fix solution (4% Para formaldehyde, 2% glutaraldehyde, 0.04% picric acid in 0.1 M phosphate buffer and 7.2 pH). After several washes in 0.1 M of phosphate buffer (pH 7.2), the samples were embedded in EPON resin mix as previously described. Further procedure for ultrastructural investigation was performed according to a previously published protocol^29^.

### Human model for conventional ultramicrotomy

Cancellous bone samples were taken from the femoral head of a patient undergoing total hip arthroplasty because of fragility fracture of the femoral neck. This investigation was approved by the local ethical committee of the Justus–Liebig–University Giessen. The bone samples were fixed at 4 °C for 24 h in Yellow Fix (4% paraformaldehyde, 2% glutaraldehyde, 0.04% picric acid) and prepared for conventional ultramicrotomy according to a previously published protocol (see Wenisch *et al*., 2003)^30^. Semithin sections (1 µm) were stained with Richardson (1% methylene blue, 1% borax, 1% azure II).

### *In vitro* osteocalcin mineralization experiments

For further and detailed description, see ref.^31^. Osteocalcin from bovine bone was purchased from Calbiochem-Novabiochem GmbH (Germany). OC was dissolved in protein buffer (pH 7.4) at a concentration of 4 mg/ml containing 10 mM sodium phosphate and 75 mM NaCl. Native bovine collagen type I, isolated from calf skin (IBFB, Leipzig, Germany) was used as collagen source. For synchronous biomineralization collagen was dissolved at a concentration of 1 mg/ml in 10 mM hydrochloric acid at 4 °C. This collagen solution was mixed with 0.1 M CaCl_2_ solution. A neutralization buffer was made by mixing 0.5 M TRIS (pH 7.4), 2 M NaCl and 0.5 M KH_2_PO_4_/K_2_HPO_4_ buffer (pH 7.4). The reaction was started by mixing the neutralization buffer with the calcium-containing collagen solution in a prewarmed (30 °C) photometer cuvette. In the case of reactions with OC, the protein solution was mixed with the neutralization buffer. The precipitated reaction products (mineralized collagen fibrils) were collected by centrifugation, washed with deionized water were freeze dried for TEM investigations. For transmission electron microscopy, we embedded samples in epoxide resin named Epofix kit (Struers GmbH, Freiberg, Germany). Ultra-thin sections of the samples with a thickness in the range of 100 nm were prepared using an ultramicrotome (Leica, Ultracut UCT, Germany – operated at room temperature) with a diamond knife (Diatome company). The thin sections were deposited on Cu-grids coated with holey carbon foils of Quantifoil with a hole diameter of 3.5 µm. The film size amounted to about 0.2 × 0.2 mm^2^.

### FIB preparation and TEM

Rat tibia samples were preserved in 4% formalin solution and stored at 5 °C prior to the FIB/TEM experiments. Formaldehyde is the widely employed fixative that has been studied and used for decades^32,33^. In order to dehydrate the samples for FIB preparation and subsequent TEM investigations, the tibia head was kept in ethanol (99.6%) for three weeks and subsequently dried at the air for 1 h^34,35^. The tibia head was crushed, and a fragment of a trabecula (approx. 0.5 mm long) was fixed onto a FIB sample holder (copper half–ring) using epoxy glue.

For TEM investigations of the trabeculae FIB thin cuts were prepared by using of a FEI Quanta 200 3D dual beam device (FEI, Eindhoven, Netherlands) with an acceleration voltage of 10–30 kV in the high vacuum mode. For electron diffraction investigations a FEI Tecnai 10 electron microscope (FEI company, Eindhoven, Netherlands) with a LaB_6_ source at 100 kV acceleration voltage was used. HR–TEM was performed by a CM 200 FEG/ Lorentz at 200 kV (FEI, Eindhoven, Netherlands) at 200 kV acceleration voltage at a nominal point resolution of 0.24 nm and line resolution of 0.20 nm. High–resolution TEM data were analyzed by using the Digital Micrograph software (Gatan company, USA). For Fourier filtering of the high–resolution TEM images the crystal reflections of the corresponding FFT were masked out with a suitable sized masking spot being well above the pixel resolution. In order to obtain large area contrast also the small angle region around the primary beam of the FFT was taken into account.

### Ethical approval and informed consent

This study was performed in full compliance with the institutional laws and the German animal protection laws. All experiments were approved by the ethical commission of the local governmental institution (“Regierungspräsidium Giessen”, permit number: 89/2009). See also Materials and Methods section.

## Results

### TEM of mineralized triple-helices of rat tibia

Light microscopy in Fig. S1a shows the general appearance of a tibia head. Fig. S1b displays a part of the tibia head sample obtained by mechanical destruction (size 5.3 × 4.4 mm). Arrows indicate the compact cortical bone as well as the porous trabecular bone where the individual trabeculae are aligned along the principal stress directions according to Martin *et al*.^36^. Figures S2a–d show SEM images of a spongy fragment of the bone interior with a size of about 2 × 1 mm. The spongiosa consists of trabeculae of about 50–100 μm thickness forming oval pores between them (Fig. S2c). These cavities have a maximum size of 200 μm, which are needed for the blood vessels and capillaries^37^. For more SEM zoom series, see Figs. S3–S5 in the Supporting Information. Focused ion beam cuts of rat tibia trabecula were prepared from broken pieces of bone, glued on a copper half ring (SEM image, Fig. S6a). TEM images of a representative FIB cut reveal that the thickness of the wedge shaped sample can be classified in three different areas: *A*, *B* and *C* (Fig. S6b). The wedge is generated by the FIB milling process and this does not mean that areas *A*, *B* and *C* represent different bone structures but it means simply the visibility of structures due to the thickness gradient. The thickness of area *A* is below about 100 nm which means that the characteristic bone structures such as the collagen striation of 67 nm is not detectable because of the thin sample. Region *B* displays well visible individual collagen fibrils with suitable contrast (Figs. S6c,f). In the thick area *C* collagen bundles with 67 nm banding are clearly seen (Figs. S6d,e), which, however, overlap in view direction and do not provide unambiguous data for interpretation. In Fig. S6c, the extension of collagen fibrils from region *B* (white circle) are shown together with the corresponding diffractogram of the white-circle area (bottom left) revealing the characteristic pattern of HAP with the *c–*axis directed almost parallel to the collagen long axis (orientational deviation ± 30°). The presence of the twisted plywood pattern is clearly demonstrated by collagen fibrils lying in plane (top) and running along the view direction (bottom) as shown in Fig. S6f ^6,38–40^. For a detailed information and discussion about the FIB technique and possible artefacts, see Supporting Information.

**Figure 1:**
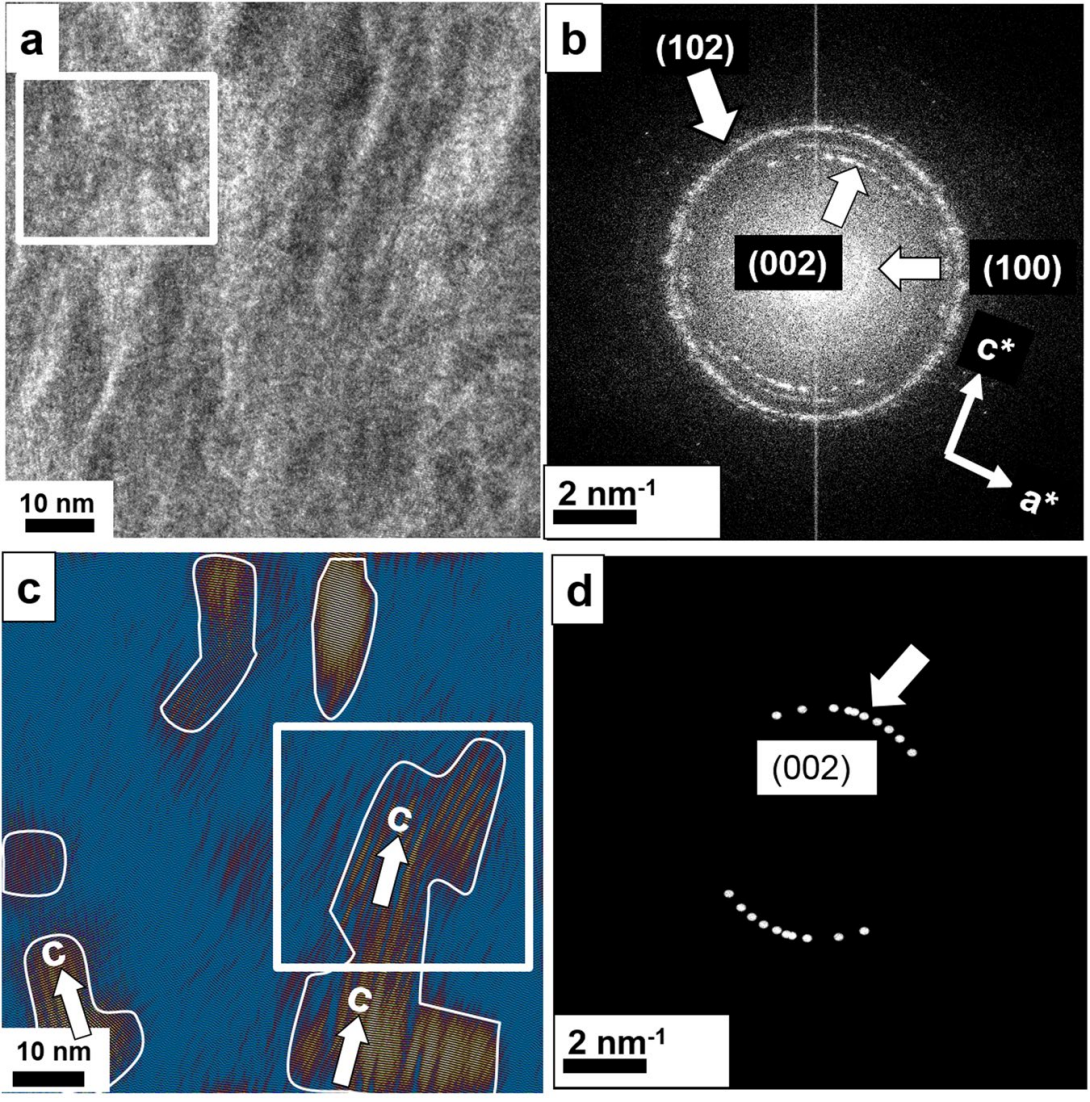
(**a**) High–resolution TEM from region B in Fig. S10b reveals a polycrystalline sample consisting of nanoplatelets of (mainly) HAP. (**b**) The presence of HAP is indicated by the respective (100) and (002) reflections of the FFT. (**c**) Fourier filtered image of (**a**). For filtering the (002) reflection of HAP was used. Nanoparticular HAP domains are marked by white frames. The striation within the platelets corresponds to 3.44 Å (002 reflection), thus the *c–*axis of HAP (see white arrows). White framed area is taken for further analysis in Fig. 2. (**d**) The mask used for Fourier filtering encompasses exclusively the (002) reflection of HAP.

A high–resolution TEM image of area *B* (see Fig. S6b) is shown in Fig. 1a and reveals that the main inorganic component consists of HAP as indicated by the strong (100), (002) and (102) reflections in the fast Fourier transform (FFT) in Fig. 1b. The (002) reflections show a fiber texture pattern with about ± 30° orientational distribution whereas the (102) reflection reveals a circumferential distribution. Thus, two different mineralization processes should be involved. Figure 1c shows the Fourier filtered image of Fig. 1a with the (002) reflection of HAP selected for the filtering process using Gaussian shaped filters with smooth edges. The micrographs clearly reveal nanoparticular HAP domains (surrounded by white lines) following the microfibrillar collagen orientation. The fibrous pattern indicates that the individual triple–helical molecules serve as template for HAP crystallization and orientation. Figure 1d shows the mask used for Fourier filtering to obtain Fig. 1c.

**Figure 2:**
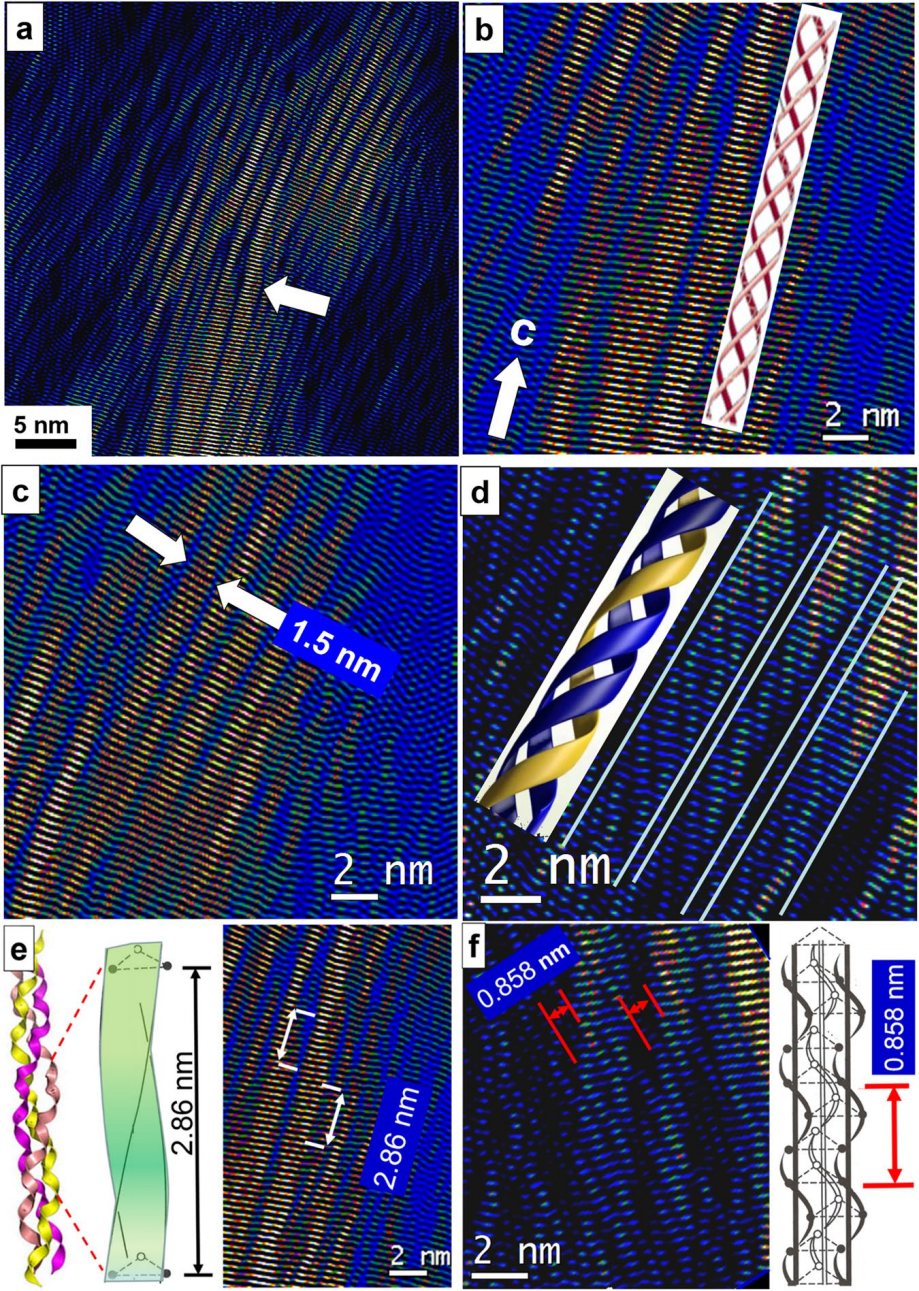
(**a**) Zoomed image of Fig. 1c (white frame). Individual mineralized fibrils are observed by Fourier filtering using the HAP (002) reflections only. Three individual alpha-chains entangling each other and forming a triple–helix are seen close to the white arrow. (**b**) The triple–helices form bundles, which are mineralized by apatite. The model beneath the experimental observed triple–helix demonstrates the idealized arrangement of the individual chains. The fine striation of 3.44 Å along the long axis of the triple–helices corresponds to the (002) reflection and thus the *c*–axis of HAP as indicated at the left bottom. (**c**) Bundle of mineralized triple–helices with the typical width of 1.5 nm of an individual fibril. (**d**) At thinner areas and at higher magnification (digitally zoomed) the winding of the alpha–chains becomes even more evident. Within the triple–helix (borders marked with white lines) the steep angle of the alpha–chain with respect to the long–axis and the spiral nature is revealed. (**e**) Comparison of predicted superstructure along the triple–helix of 2.86 nm (left) and supercoiling as read out from the TEM high–resolution (right). (**f**) Indication of winding period of alpha–chains. The periodicity along the triple–helix amounts to 0.86 nm and is evoked by the alpha–chain helix indicated on the model at the right.

In order to obtain more detailed information on the calcification scenario of the triple-helical collagen molecules, the filtered image of Fig. 1c (white frame) using the (002) reflection of HAP was digitally zoomed. In Fig. 2a mineralized bundles of collagen fibrils are shown where individual triple–helices can clearly be distinguished (see *e*.*g*. white arrow). In the further zoomed area at the white arrow of Fig. 2a even the individual alpha–chains wound around each other become visible (Fig. 2b). The model of a triple–helix is added to demonstrate the ideal case. Figure 2c displays another assembly of mineralized triple–helices selected from top right in Fig. 2a. The width of 1.5 nm of the fibrils is indicated by arrows and corresponds to the expected value. Figure 2d shows triple–helices at high magnification where the winding of the alpha–chains is clearly resolved. As already shown in Figs. S6c and 1c, the *c–*axis of HAP runs almost parallel to the long axis of the collagen molecules (fibrils). The triple–helices form strands of about 1.5 nm in diameter, which are subsequently mineralized by apatite. The *c–*axis of apatite gives rise to a fine striation pattern of 3.44 Å along the long–axis of the triple–helix. In Figs. 2e,f the predicted periodicities along the triple-helical chain are compared showing accordance with our TEM experiments. By X–ray fiber diffraction on tendon a supercoiling of 2.86 nm along the triple–helix was observed, which we were able to confirm by direct imaging (see Fig. 2e)^4,41–44^. On short chain model peptides a different superstructure of 2.0 nm was found^4,45^. The alpha–chain helix periodicity amounts to 0.86 nm along the triple–helix as indicated on the model in Fig. 2f, right (4). In the Fourier filtered high–resolution the same winding periodicity was revealed (Fig. 2f, left).

### TEM of mineralized triple-helices of human femur

**Figure 3:**
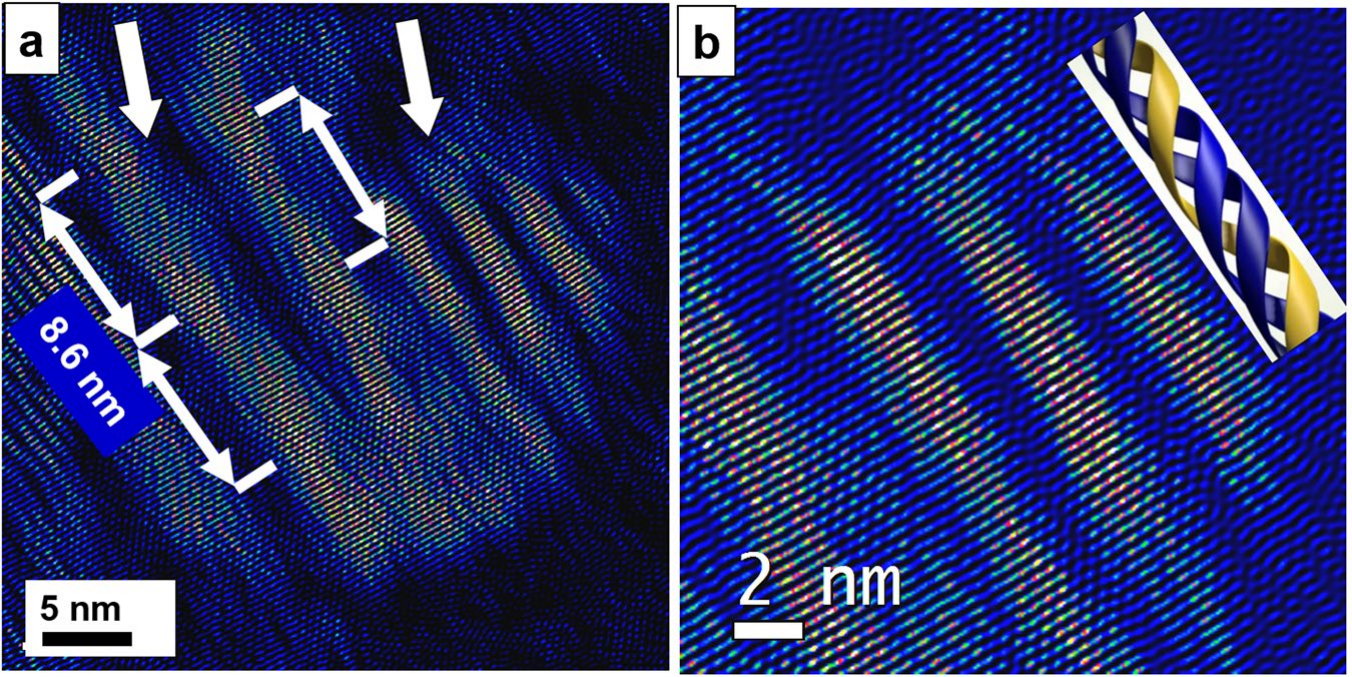
(**a**) The occurrence of mineralized triple–helices is not confined to rat bone only but is also found in human hard tissue: Assembly of mineralized collagen microfibrils (left arrow) and triple–helices (right arrow) of human bone. (**b**) Zoomed area of (**a**) displaying mineralized triple–helices situated at the right together with the model of a triple–helix. The winding of the alpha–chains within the fibril is clearly imaged.

In order to ensure that the observed structures are not artefacts caused by FIB preparation we also investigated human bone (femur), which was prepared by conventional ultramicrotomy (see Materials and Methods)^30,46^. For SEM and light microscopy images see Supporting Information Fig. S7. With these additional investigations we support the existence of mineralized triple–helical collagen fibrils and microfibrils as general phenomenon and exclude that they only occur in animal hard tissue. Microfibrils are assemblies of five triple–helices and amount to 4.5–5.0 nm in diameter. They represent the subsequent step within the hierarchy of collagen bundling following the triple–helix fibril^25,47,48^. Figure 3a displays an assembly of mineralized microfibrils (left arrow) and triple–helices (right arrow) in human bone. Along the microfibril a periodicity of 8.6 nm is observed, which is in accordance to former reports on rat tendon and lamprey notochord by Orgel *et al*.^49,50^ or even much earlier work of Okuyama *et al*. from 1977^51^. The magnified image taken from right top of Fig. 3a shows triple–helical molecules where the winding of the alpha–chains within the fibril is observed (Fig. 3b). In spite of the orientational deviation between the individual fibrils the apatite component manages to form an overall calcification of the biological template by adopting these small differences of misorientations along its *c–*axis. This kind of intergrowth scenario of the bio–composite was already described in our former atomistic simulations on the nucleation of fluorapatite (FAP) by collagen triple–helices^52^ and bears strong resemblance to a mesocrystal where the bulk material is formed by uniformly oriented building blocks^53,54^. Motifs of the apatite crystal structure are fixed and stabilized by the triple–helix in a way that nanocrystals (nanoplates) can grow from these nuclei in an orientation with their hexagonal *c–*axis direction almost parallel to the long axis of the triple–helix. Additional TEM experiments on the biomimetic model–system FAP/gelatine under *in vitro* conditions confirm the templated and crystallographically oriented growth of apatite at triple–helical collagen bundles and thus the mineralisation of collagen at the atomic level^55^.

### TEM of osteocalcin in rat tibia

**Figure 4:**
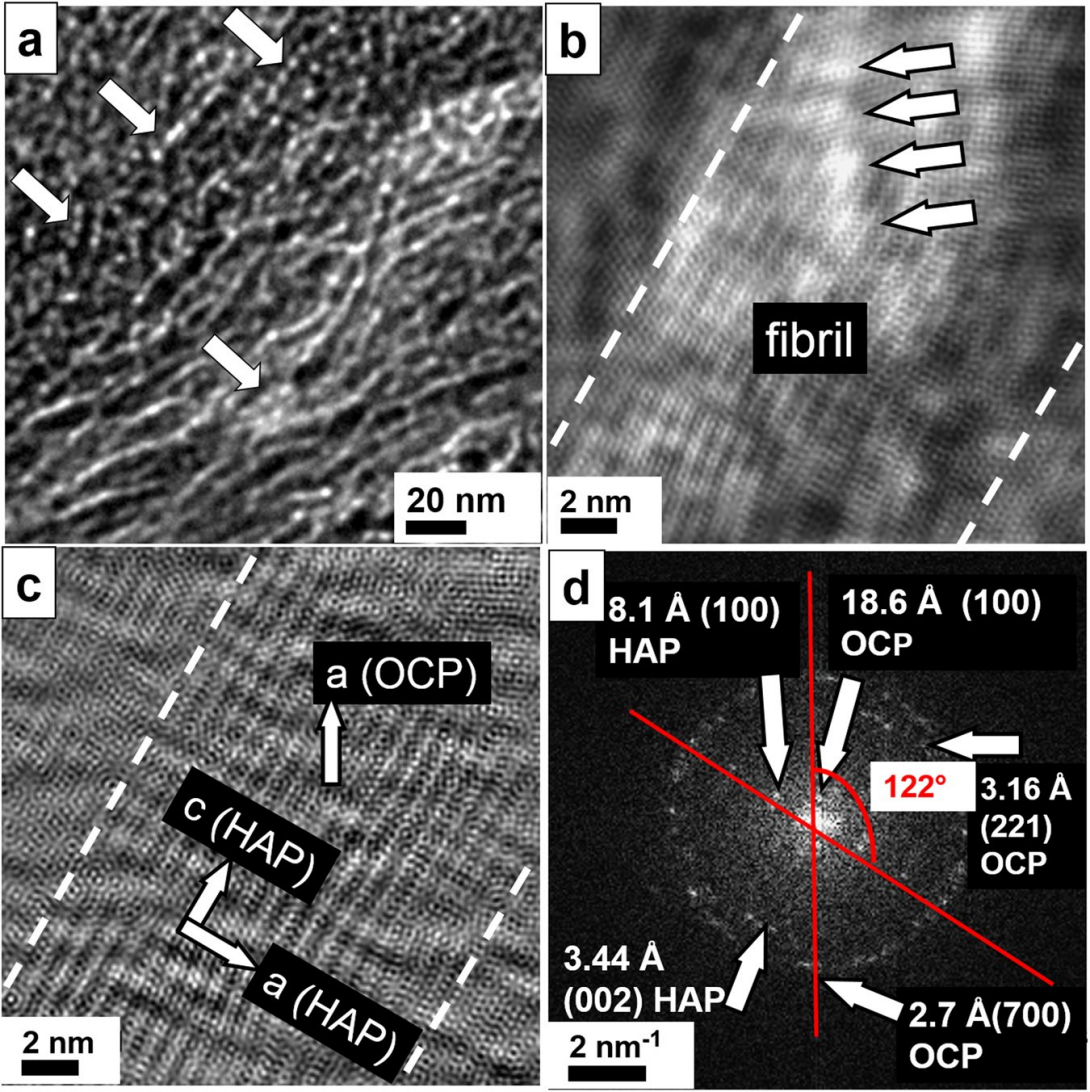
(**a**) In overview images, pearl necklace-like strings were observed in the sample. The strings consist of beads, which are about 2 nm in size. (**b**) High–resolution of the strings: Small angle filtered TEM image at higher resolution showing only the large spacings caused by the organic components. Spherical structures with about 2 nm in diameter covering the fibril and forming a pearl necklace string (white arrows). (**c**) Fourier filtered image using the (002) and (100) reflections of HAP, and the (100) reflection of OCP. OCP with [0–12] orientation is present along the osteocalcin pearl necklace strings (see (**a**)) whereas HAP extends straight along the collagen fibril. (**d**) Fourier transform in the small angle region clearly reveals the presence of OCP besides HAP (see arrows).

In overview images (Fig. 4a), pearl necklace strings were observed. For further HR–TEM analyses, the area just above the mineralized fibril shown in Fig. 2a was selected. The low–pass filtered HR–TEM micrograph was taken by additionally using the crystal reflections of HAP for the filtering procedure. In the filtered micrograph (Fig. 4b) spherical structures of about 2 nm in diameter (white arrows) are stringed in line. Based on their shape and dimensions, these objects are identified as a non–collagenous protein, most probably as osteocalcin in its folded state, covering the fibril^10–13^. OC has an exceptional low molecular weight of less than 6 kD being by far the lowest among the non-collagenous proteins occurring in bone. Additionally, its high content in calcified tissues makes him the most probable candidate for the observed structures. In Fig. 4c only the crystal reflections without the small angle region corresponding to organic structures were selected for filtering. The Fourier transform not only shows the strong reflections (002) and (100) of HAP besides other mixed reflections, but also the strong (100) reflection of OCP corresponding to 18.6 Å (Fig. 4d). This pattern clearly indicates the presence of both phases, HAP and OCP. The HAP phase mainly extends along the collagen bundle, whereas OCP is preferably detected along the pearl necklace ordering of the osteocalcin spheres with [0–12] crystal orientation.

**Figure 5:**
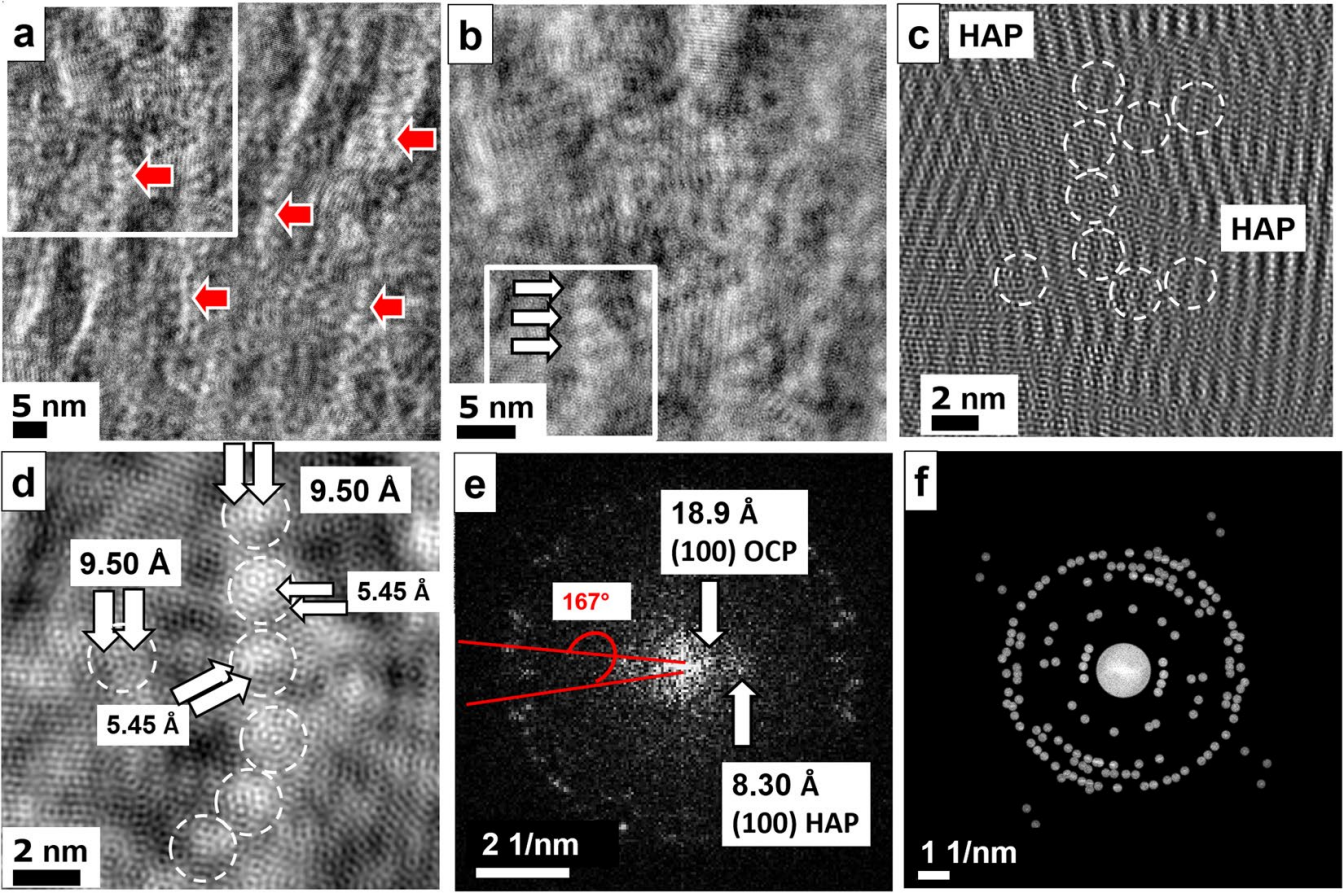
(**a**) Filtered image of the white framed area in Fig. 1a. For filtering all crystal reflections of HAP and OCP together with the small angle region corresponding to organic components (collagen fibrils and osteocalcin structures) were considered. The image appears highly structured by collagen fibrils superimposed with pearl necklace-like arrangements (red arrows) along the collagen fibrils indicating the presence of osteocalcin. (**b**) Magnified view taken from the top left region marked by a white frame in (**a**). One of the osteocalcin chains is marked by arrows. (**c**) High-resolution image where only crystal reflections were taken into account for filtering. The osteocalcin beads are clearly visualized between the HAP crystals appearing as vertical bright-dark stripes. (**d**) Magnified area of the bottom left part of (**b**) (white frame) showing the osteocalcin chain and single osteocalcin individuals close by (white circles). The preferred binding sites of osteocalcin to the Ca sites of the OCP/HAP structures amount to about 9.5 Å (Ca…Ca distances along *b*-axis within the OCP (100) plane) as well as along *b*-axis within the HAP (100) plane. In addition, binding distances of 5.45 Å are observed. (**e**) FFT of (**c**) with osteocalcin chain revealing the presence of OCP and HAP. (**f**) Mask used for Fourier filtering including all crystal reflections and small angle region. In (**c**) only the crystal reflections were taken into account without the small angle area.

Assuming that the globular structures shown in Figs. 4a,b are representative for the presence of osteocalcin, we investigated the sample in order to find further indications for the appearance of similar patterns close to mineralized collagen fibrils, which could be a hint for osteocalcin – Ca^2+^ complexes being involved in the crystallization scenario. In order to visualize the non–collagenous protein, the small angle region also normal to the fibril direction was taken into account together with the crystal reflections of HAP and OCP. In Fig. 5a the filtered image taken from the white frame area in Fig. 1a shows a highly structured substrate consisting of collagen fibrils and pearl necklace strings (see red arrows). The globular structures with diameters of about 2 nm most probably indicate the presence of the non–collagenous protein osteocalcin. Figure 5b displays the digitally zoomed top left region of Fig. 5a (white frame) obtained by using all available crystal reflections together with all spacings containing the small angle region in order to extract the complete structural information. The imprints of the osteocalcin structure together with the collagenous fibril matrix give rise to a complex mineralization pattern. Globular assemblies (arrows) vertically aligned with a chain–like structure and a periodicity of about 2.5 nm along the strings become evident as marked by the white triple arrow. Figures 5c,d are the zoomed representations of the lower white frame region in Fig. 5b. In Fig. 5c only the crystal reflections of HAP and OCP were considered for filtering. The vertical strong bright-dark stripes are crystal lattices of HAP. Between the HAP crystals the pearl necklace structure and additional other globular structures are revealed. At atomic resolution we are able to identify the preferred binding sites of the osteocalcin molecules (white circles) with typical spacings from one calcium site to the neighboring site of about 5.45 Å and 9.5 Å corresponding to *b*_(OCP)_ = 9.52 Å and *a* = *b*_(HAP)_ = 9.42 Å, respectively (Fig. 5d, see arrows)^23,24^. Fast Fourier analysis of the crystal reflections along the osteocalcin chain (Fig. 5e) evidences the occurrence of OCP with typical reflections such as (100) at 18.9 Å besides HAP (100) at 8.30 Å. Figure 5f shows the mask used for Fourier filtering for Figs. 5a,b,d where crystal reflections as well as small angle region were included. For mineralized OC the 3.17 Å reflection corresponding to (102) HAP and (221) OCP are the most important besides 2.80 Å (211) HAP and (530) OCP reflections. Less intense reflections such as 2.64 Å (202) HAP and (711) OCP or 1.83 Å (213) HAP were also taken into account. Crystallographic relations between HAP and OCP are discussed in more detail in the chapter for discussion.

**Figure 6:**
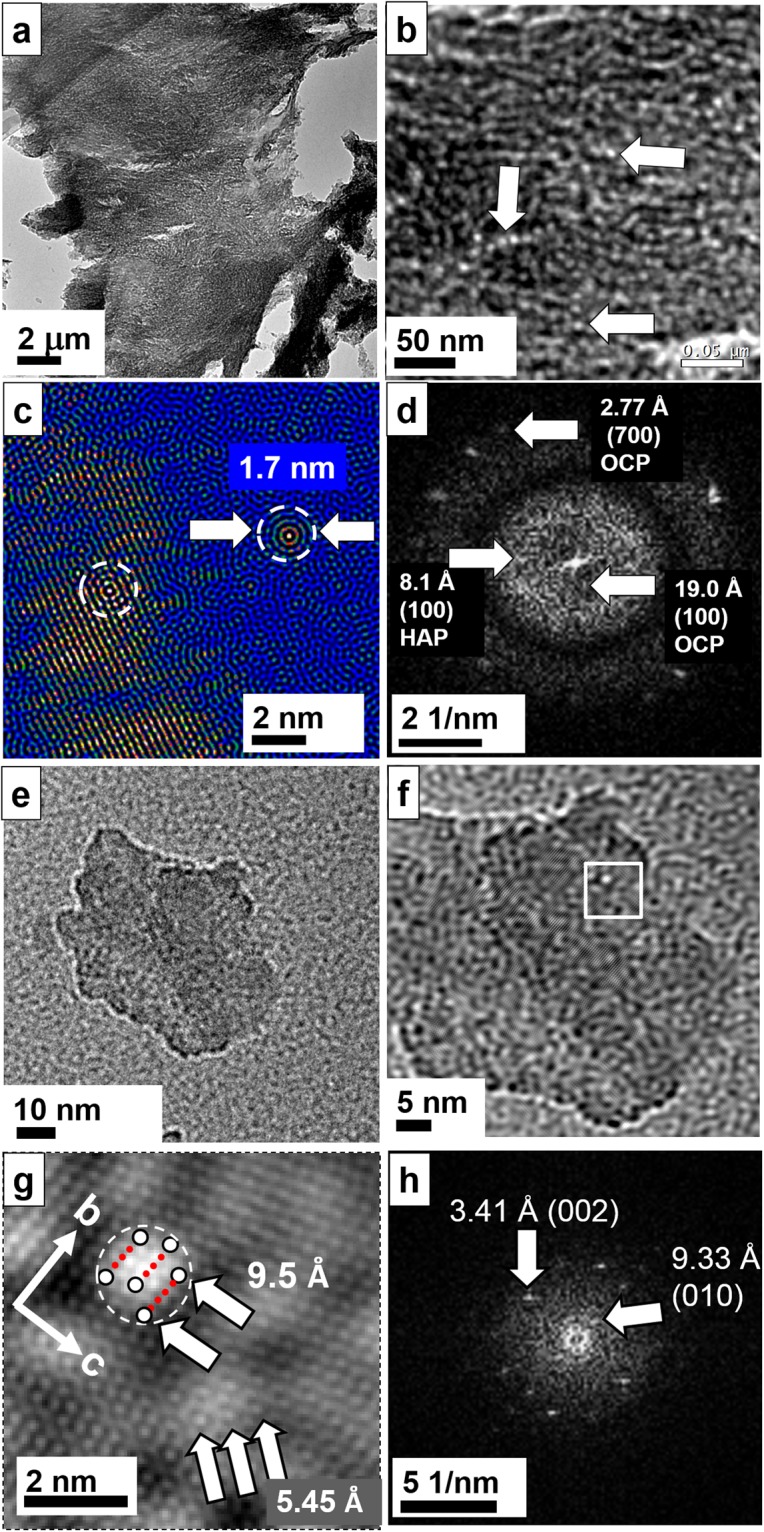
Rat vertebra prepared by conventional ultramicrotomy. (**a**) Overview image. (**b**) Magnified image displays necklace pearl strings and individual osteocalcin molecules (see arrows) in the sample. (**c**) At high–resolution, the OC protein is visualized as concentric sphere (see dotted areas). One OC molecule is situated directly on the HAP/OCP crystal on the left whereas the other is near the crystal. (**d**) The FFT clearly indicates the presence of octacalcium phosphate in connection with OC. (**e**) Single crystal platelet of OCP in (100) orientation from rat trabecular bone. (**f**) The small angle filtered image indicates an additional globular structure (nearly close packing) consisting of the non–collagenous protein osteocalcin. (**g**) The Fourier filtered HR–TEM image shows the OCP lattice ((100) plane) together with assumably osteocalcin or/and another NCP (one molecule indicated by a white circle together with the Ca binding sites of about 9.5 Å distance). The region of interest is marked by a white frame in (**f**). (**h**) Fourier transform of (**g**) showing the presence of OCP.

Diamond cuts may provide thinner samples and areas of interest, which are significantly larger up to several mm in contrast to 5–10 μm obtained for FIB cuts. By investigating conventional diamond cuts of rat vertebra one should be able to confirm the FIB cut results and thus exclude artefacts caused by the gallium beam milling. For further details about the diamond thin cut preparation (ultramicrotomy) of the sample, see Materials and Methods and citation no.^29^. Overview TEM images of rat vertebra are presented in the Supporting Information Fig. S8. Figure 6a shows an overview image of the vertebral rat bone. At higher magnification, pearl necklace chains of the OC become visible (Fig. 6b, see arrows). The size of the nodules of about 2 nm fits to the size of OC molecules. The same structures were detected in the FIB cut (see *e*.*g*. Figs. 4a, 5a). The Fourier filtered high–resolution image reveals that the nodules are in fact OC particles. They show a circular morphology with 1.7 nm diameter (Fig. 6c). The corresponding FFT indicates the occurrence of OCP at the same time (Fig. 6d). Next to densely packed nanofibrils almost fibril free regions can be found. In the absence of collagen fibrils, we observed larger nanocrystalline platelets of OCP (Fig. 6e). In Fig. 6f a small angle Fourier filtered micrograph of an OCP platelet from trabecular bone is displayed with a diameter of about 50 nm showing globular structures which are assumed to represent osteocalcin and may also involve also other non–collagenous (folded) proteins. The high–resolution image where all OCP reflections were considered for filtering (Fig. 6g) clearly displays the distribution of osteocalcin and possibly other NCP on the OCP platelet with Ca binding sites of 9.5 Å distance perfectly fitting to the *b-*direction of the OCP crystal structure. In fact, the Fourier transform of the crystal lattice (Fig. 6h) shows the presence of OCP viewed from the [100] zone (OCP platelet with (100) orientation).

### *In vitro* mineralization experiments of OC in the presence of collagen

**Figure 7:**
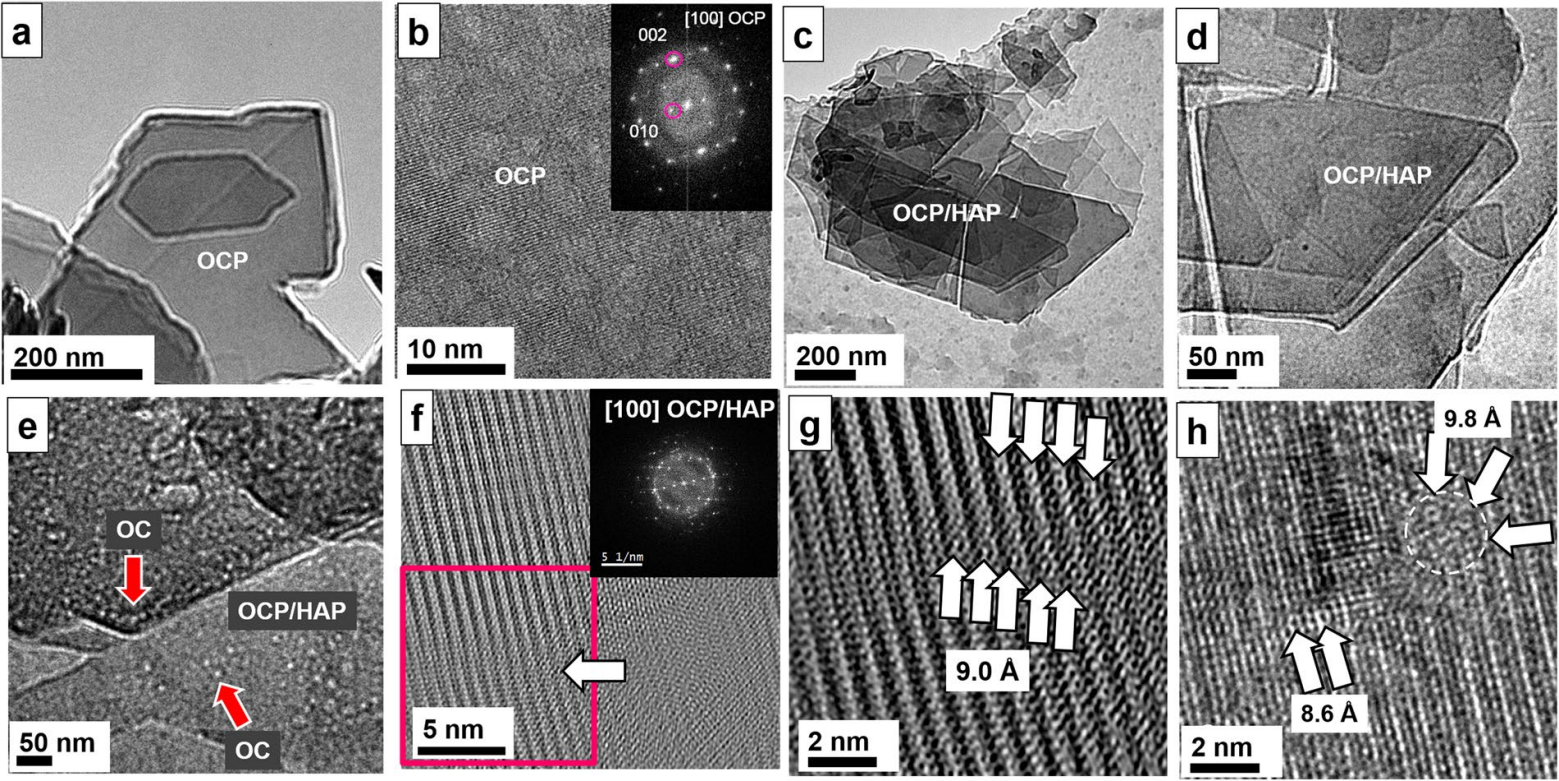
*In vitro* mineralization of OC. (**a**) Occasionally, pure OCP platelet formation (several 100 nm in size) was observed under the influence of OC. (**b**) High-resolution of the crystal shows the presence of OCP, [100] zone. (**c–e**) Zoom series of OCP/HAP plates. (**e**) At higher magnification, beads of OC are imaged covering the surface and the edges of the plates. Pearl necklace formation is dominating but also isolated individuals are revealed, see arrows. (**f**) Filtered high-resolution with corresponding FFT, [100] zone. At bottom left, the crystal lattice shows a discontinuation (arrow) due to the presence of OC. (**g**) Magnified view of red marked area in (**f**). In high-resolution the disruption zone amounts to 2 nm corresponding to the diameter of a pearl necklace structure. Individual binding sites (arrows) of the carboxylic groups are identified bearing strong resemblance to the binding sites identified for bone. (**h**) Non-filtered high-resolution of another disruption zone of crystal lattice. At right top, a 2 nm sized OC is imaged containing three binding sites marked with three arrows and a dotted white circle.

In order to prove that OC is the acting agent, which we identified among the non-collagenous proteins in bone we carried out also a number of experiments *in vitro*. We investigated *e*.*g*. osteopontin^56,57^, phosphoserine^57^ and osteocalcin^31,56^. The *in vitro* mineralization experiments in the presence of collagen and OC resulted in pure OCP (Fig. 7a) as proved by high-resolution TEM (Fig. 7b) and OCP/HAP plates (Figs. 7c–h). For details, see Materials and Methods^31^. Figures 7c–e show a zoom series of the HAP/OCP plates. Beads with 2 nm diameter are observed on the surface of the plates either organized as pearl necklaces mainly at the edge of the plates (see arrow at top) or as single particles (Fig. 7e). Figure 7f shows the Fourier filtered high-resolution image together with the FFT. On the left bottom, the lattice is discontinued due to a pearl necklace arrangement of OC, see arrow. The zoomed high-resolution image in Fig. 7g shows the disruption zone of about 2 nm in width in detail. A continuous line of six binding sites on the atomic scale could be identified with a regular periodic distance of about 9 Å, see arrows. In Fig. 7h (non-filtered high-resolution) the crystal lattice is disturbed by single particles. On the right top, the crystal lattice is interrupted by a spherical particle with three binding sites (marked by arrows). On the left bottom, another singular OC is shown with the binding sites indicated by arrows. The similarity of the structures observed in the *in vitro* experiments is striking to the examples shown before on bone as well in overview as well in high-resolution. In both cases, we see bead structures with the tendency to form pearl necklaces and we can identify in both *in vitro* and *in vivo* the γ-carboxylated glutamic acid binding sites of OC with similar distances of 5.45 and 9–10 Å. Besides this, in both cases the formation of OCP is preferred due to the high acidic binding sites of OC.

### TEM observation of osteocalcin in human femur

**Figure 8:**
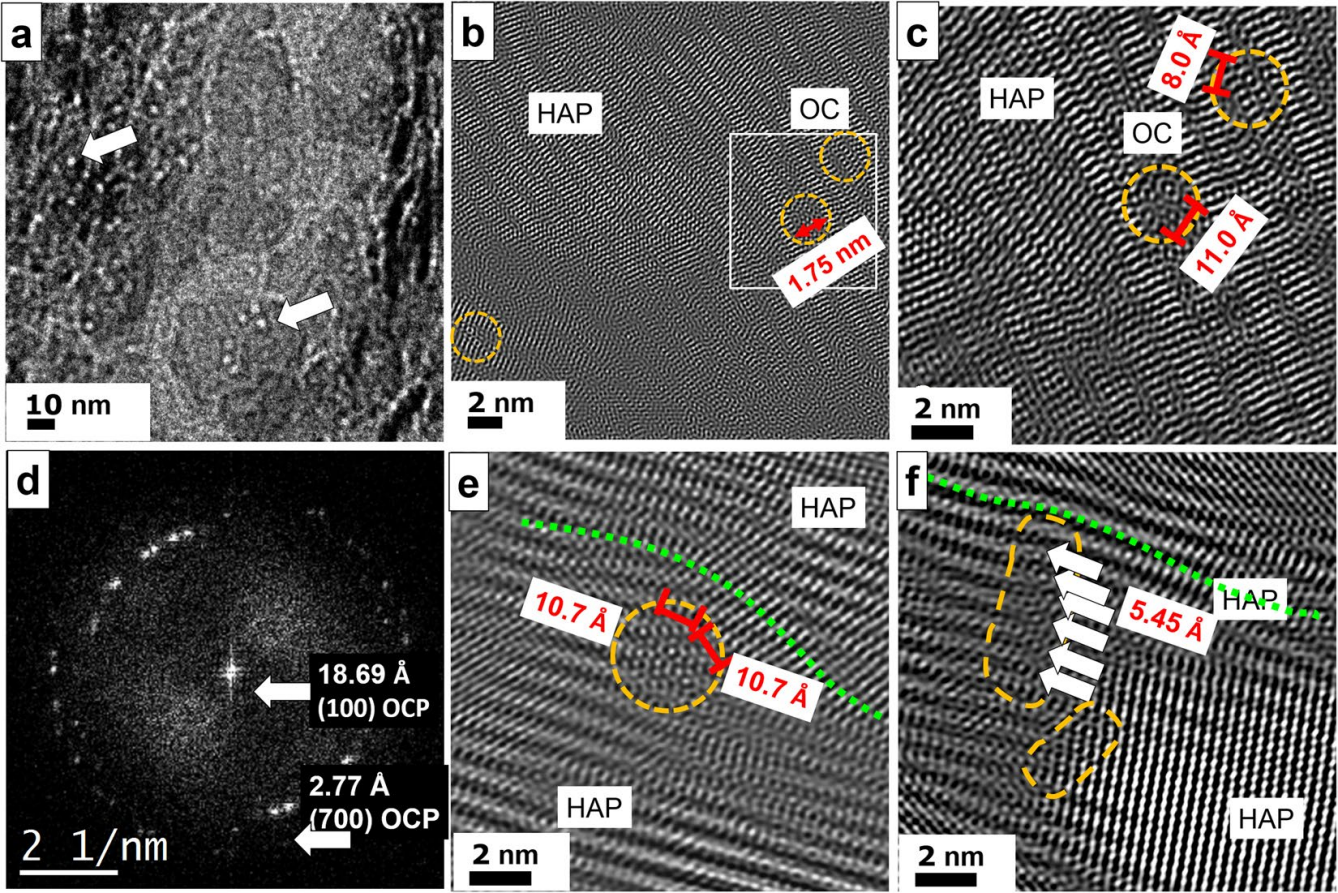
TEM of conventional ultrathin diamond cuts of human femur. (**a**) Overview image with pearl bead strings of 2 nm diameter indicating the presence of osteocalcin, see arrows. At the center bottom a 50 × 50 nm^2^ large HAP crystal plate is imaged with OC particles on its surface resembling to white pearl bead structures suppressing the HAP growth normal to the plate plane. (**b**) The Fourier filtered high–resolution image displays a massive HAP crystal at the top of the micrograph. At certain regions, the crystal lattice is disturbed due to the presence of OC (see orange circles at right). (**c**) At higher magnification a gap within the crystal plate is revealed corresponding to the size of OC proteins. Even the binding sites are resolved which appear as small circles with pronounced dots in the center (distances of 11 and 8 Å). (**d**) The FFT of the high–resolution image show reflections at small angle corresponding to 18.69 Å, thus proving the presence of OCP. (**e**) OC is also found between HAP crystals with different orientations serving as delimiter. The insertion of OC evokes mechanical stress and leads to remarkable lattice distortions of the upper crystal (green dotted line). The binding sites within OC show 10.7 Å distance from each other. (**f**) The massive crystal plate on the right is confined by a string of OC particles marked in orange. At least six binding sites (see arrows) are arranged in a row.

Furthermore, conventional diamond cuts of human femur were investigated in order to prove the occurrence of octacalcium phosphate/ osteocalcin nanocomplexes as a general mechanism preventing massive crystallization of collagen. For further details of the thin cut preparation see Materials and Methods^30^. In Fig. 8a center, two HAP crystals are shown whereas at the left and right side a dense covering of OC is revealed (left arrow). The HAP crystal at the bottom is partially covered by OC appearing as white dots in the image (right arrow). In the Fourier filtered high–resolution image (Fig. 8b) at top a HAP crystal is imaged. At certain positions the crystal order is disturbed (orange circles) and even gaps within the crystal are observed with a width of about 1.75 nm corresponding to the size of the OC molecule. At higher magnification (Fig. 8c), the binding sites of the osteocalcin become visible as tiny circles with a dot in the center bridging a distance of about 11 Å. The corresponding FFT (Fig. 8d), indicates the presence of OCP with the typical distance of 18.69 Å corresponding to the (100) lattice plane. In Fig. 8e a typical situation is shown where osteocalcin particles (orange circle) are situated between two HAP crystals (top and bottom of image) and thus forming a borderline between them. The upper HAP crystal is mechanically deformed due to the insertion of OC (see green dotted line). A similar process is recorded at vicinal area (Fig. 8f). The massive HAP crystal at the right bottom is confined in its growth by the OC particles (orange circles) in the center. The fine white stripes inside the plate indicate the (002) lattice plane corresponding to 3.44 Å. At the top, another HAP crystal with a different orientation reaches the OC particles. The broad stripes indicate the (100) lattice planes of 8.1 Å, which are slightly bend due to the blocking behavior of OC.

### Beam damage TEM experiments

In order to test and to determine the stability of the FIB cut sample, we performed time–dependent HR–TEM electron irradiation experiments at harsh conditions (Supporting Information Figs. S9a–c and S10a–c) of the area presented in Fig. 5b. The applied electron dose rate in Figs. S10a–c amounted about 2000 electrons per square Angstrom and per second, which is double of the dose rate used in the Supporting Information Figs. S10a,b and much more accumulated dose, which exceeded ~25 (Fig. S10b) and ~110 times (Fig. S10c) the actual applied dose. After several recording and focusing sequences, the crystal structure is still preserved as indicated by the corresponding Fourier transforms of the high–resolution images (Figs. S10a–c, bottom). Only after three minutes persistent irradiation and about 493.500 e/Å^2^ accumulated dose the crystal showed changes, see Fig. S10c, arrow, whereas the FFT still seems not to be affected, keeping the resolution of 1.48 Å. Therefore, for this area we exclude that under the actual recording conditions the crystal is already affected. In order to prove that the observed structures are not simply beam damage artifacts, we recorded high–resolution images with high electron doses. In this way, we take into account damage that is caused by focusing the sample and for recording the images at high magnification. An exhaustive discussion of the beam damage effects together with references is presented in the Supporting Information. Additionally, high–resolution TEM of a biomimetic apatite–gelatine composite is shown in the Supporting Information Fig. S11. The triple–helix is clearly imaged at an applied dose rate of about 770e/Å^2^s without hints for beam damage of the buried protein structure within the apatite matrix^58^.

## Discussion

Nowadays, bone structure is still a highly investigated topic and till now a lot of questions has to be answered, *e*.*g*. such as the relationship between the mineral component of bone and the organic component collagen. McNally *et al*. and Schwarcz showed on FIB cryo milled cortical human bone that HAP platelets form quasi–circular walls wrapping around the fibrils confirming a model that the majority of mineral in bone resides outside the collagen fibrils (33 vol.% of bone) instead of the gap zones (corresponding to 12 vol.% of bone)^59,60^. The (chemical) composite nature of bone on the molecular level is already accepted. For example, Schwarcz name the apatite platelets “mineral lamellae” and describes that these platelets are composites with collagen, which bend around the collagen fibril. He infers that, inside the lamella, these crystals are held in place by one or more molecular species which, when oxidized, liberate apatite single crystals^60^. In our study, investigations on rat and human bone show that the collagen fibril itself is also mineralized (Figs. 2, 3). In the case of the rat FIB cut, the mineralized platelets are about 10–12 nm wide and 22–25 nm long, see Fig. 1c. We assume that the interfibrillar platelets are also present but may have other dimensions. In literature, early TEM measurements on cortical compact bone gave following sizes: length 50 nm, width 25 nm, 10 nm thickness^61–63^. These sizes were confirmed for cow, chicken, mouse, rat and fish. The smallest of them had irregular roundish shape and with a thickness of 1.0–1.2 nm. The maximum length of animal platelets amounted to 25 nm and maximum width to 12–15 nm. Fish bone proved to be larger: length 37–50 nm and width 15–20 nm. Further measurements on mineralized turkey tendon supported these values^64–68^. All experiments up to now show apatite as only mineral phase in bone of vertebrates. Nevertheless, theoretical and thermodynamical investigations lead to the assumptions that the initial nuclei should be octacalcium phosphate^19^ or an amorphous calcium phosphate^69–73^. Later, these precursor clusters are dissolved in water or are transferred by a solid-state reaction into apatite. Until today, morphology, size and 3D relationship to collagen is a strongly debated topic^74–78^. In the moment, it is assumed that for healthy bone the apatite crystals are platelet like with 15–150 nm length, 10–80 nm width and thickness 5–2 nm. An average for human bone amounts to 50 × 25 × 10 nm. For osteoporotic bone, contradictory sizes are reported being either smaller, same or even larger than healthy bone platelets^79^.

**Figure 9:**
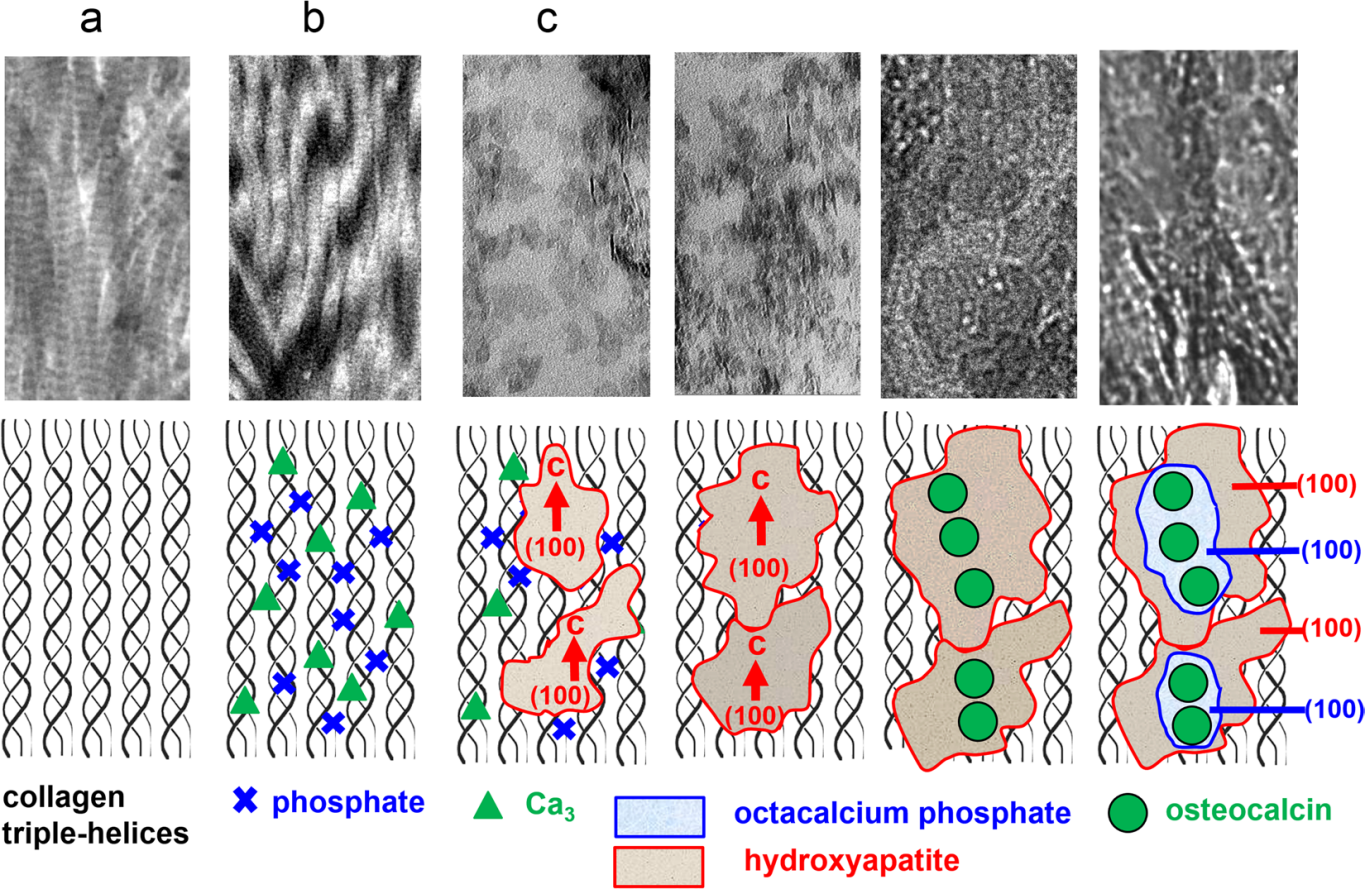
Possible scenario and schematic sketch of calcification steps in and at a collagen fibril derived from snapshots. (**a**) Part of a collagen fibril consisting of protein triple–helices in parallel arrangement. (**b**) Pre–orientation of Ca_3_–triangles (Δ) and phosphate groups (x) as precursor–scenario containing motifs of the hydroxyapatite (HAP) crystal structure, pH > 6.5. (**c**) Formation of HAP nanocrystals (red) with platy habit (100) and the *c*–axis direction in parallel orientation to the fibril extension. (**d**) Growth of HAP nanoplatelets. (**e**) Attachment of osteocalcin (OC) on the composite surface (collagen fibril and HAP nanoplatelets). Lowering the pH (<6.5). (**f**) Epitaxial growth ((100) HAP/(100) OCP) of octacalcium phosphate (OCP, blue) on HAP (red).

Rat trabecular bone represents a highly dynamical system with a high rate of remodeling with time. Thus, by TEM investigations various snap shots of different stages of bone development are recorded, which are representative for a restricted area only. Therefore, a complete and comprehensive mineralization scheme is hard or impossible to deduce from snapshots alone. Nevertheless, we try to propose the following possible crystallization scenario at the atomic scale deduced from the high-resolution images. First, likely the individual triple–helices within thinner fibrils are mineralized along the *c–*axis. Supposable, this scenario starts with binding of Ca^2+^ and (PO_4_)^3−^ ions to the charged functional groups along the fibril. By further diffusion of ions small HAP plates are formed. When these first seeds meet each other, ripening takes place giving rise to massive crystallization (Fig. 9). This situation at the nanoscale bears a strong resemblance to the formation process of mesocrystals where oriented attachment of smaller building blocks is required to achieve a uniform orientation in the bulk phase^53,54^.

The functionality of osteocalcin in mineralization has been interpreted as a process–directing agent for the intrafibrillar nucleation of HAP crystals in the type I collagen macrofibrils^16,80^. The collagen fibril can be regarded as the structure-directing agent^81^. Particular motifs of amino acids initiate the crystallization of HAP. This assumption is supported by atomistic computer simulations of the interaction of the calcium and phosphate ions with a model molecule built of three (Glycin–Proline–Hydroxyproline)_12_ polypeptide strands arranged in a triple–helical structure. (Gly–Pro–Hyp) is the dominating motif of amino acids in the tropocollagen filament^52^. As shown in the calculation, the calcium ions form ionic bonds to the oxygen atoms of carbonyl groups of the polypeptide backbone and to the side chains of proline and hydroxyproline, whereby the global shape of the triple–helix is not affected^52^. The Coulomb repulsion of the calcium ions bound at the backbone causes a straightening and an additional stiffening of the tropocollagen molecule.

Reznikov *et al*. showed that the lamellar unit of human cortical bone is a composite of ordered and disordered material of mineralized collagen fibrils with different degrees of alignment^82^. Trabecular bone is in fact as organized as cortical bone^83^ but contains overlapping lamellar packets instead of cylindrical osteons^6^. The lamellar structure of human trabecular bone is a composite of ordered and disordered materials and is very similar to the compact bone lamellar structure at a scale of micrometers.

**Figure 10:**
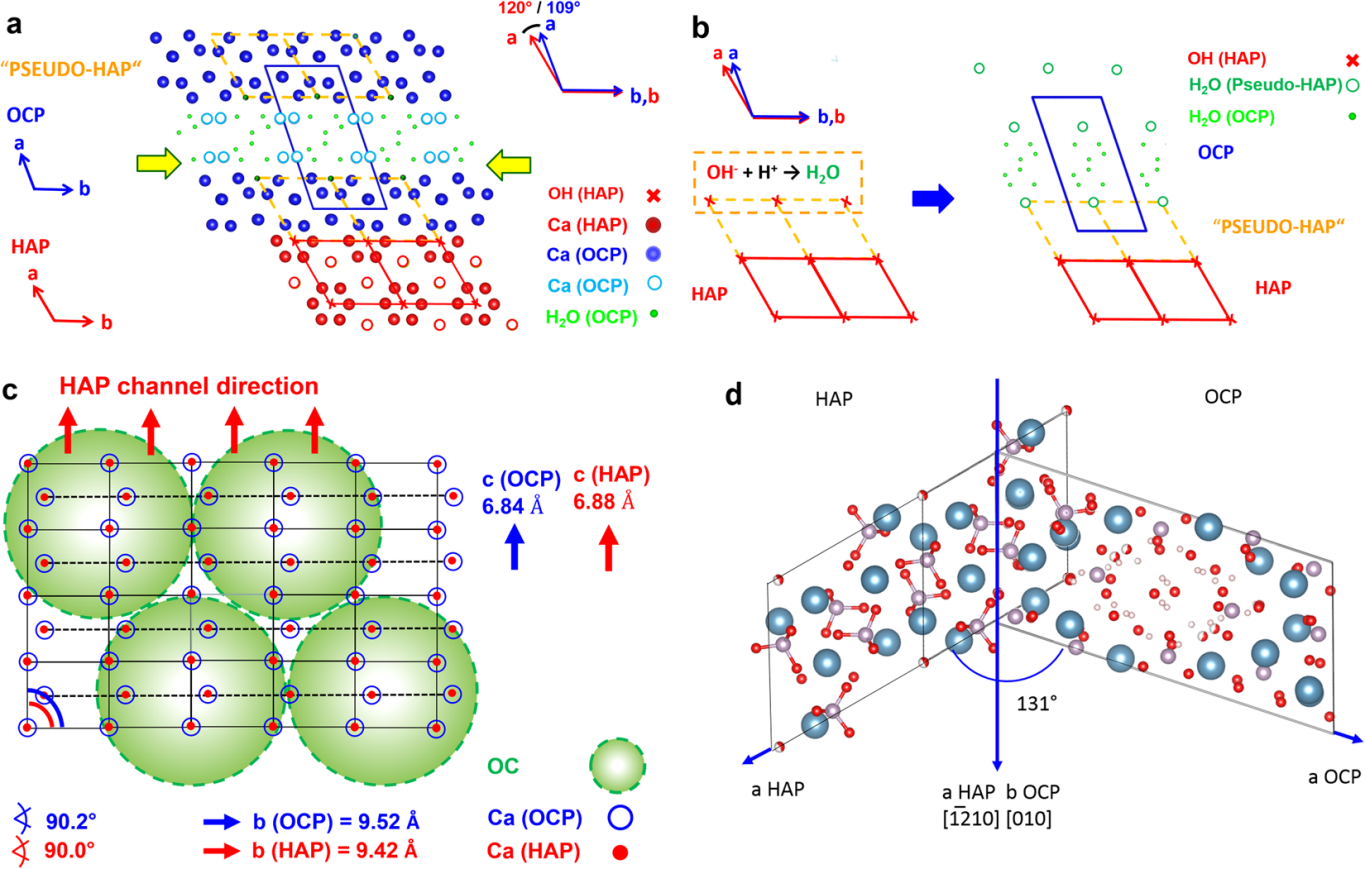
(**a**) Structural relations between octacalcium phosphate (OCP, Ca_8_(HPO_4_)_2_(PO_4_)_4_ ·5H_2_O, *P*-1, *a* = 19.69 Å, *b* = 9.52 Å, *c* = 6.84 Å, *α* = 90.2 °, *β* = 92.5 °, *γ* = 108.9 °)^23^ and hydroxyapatite (HAP, Ca_10_(PO_4_)_6_(OH)_2_, *P*6_3_/*m*, *a* = *b* = 9.42 Å, *c* = 6.88 Å, *α* = *β* = 90.0 °, *γ* = 120.0 °)^23^. Projections along c* (OCP) and *c* (HAP). Atoms restricted to Ca (OCP: blue, HAP: red), H_2_O (OCP: green), OH (HAP: red cross). Apatite–like layers in OCP (“Pseudo-HAP”) indicated by “unit cells” (broken lines in yellow). The epitaxial relation (100, OCP)/(100, HAP) is close to ideal, see schematic sketch in (**b**): Distribution of Ca- sites (red/blue) on (100, OCP)/(100, HAP) as present (for instance) on (100, OCP ≙ “pseudo–HAP”) close to the H_2_O–containing layers of OCP (see yellow arrows in (**a**)). Green circles: Possible areas (diameter of about 2 nm) for attachment of folded osteocalcin molecules. See TEM images in Figs. 4–7. Ca atoms (HAP, OCP) in plane. (**c**) Schematic sketch of the epitaxial growth of OCP (blue) on HAP (red) by lowering the pH (<6.5) under the influence of osteocalcin (OC). Formation of H_2_O molecules by reaction of OH^−^ (from “surface channels” on (100) in the HAP crystal structure) with H^**+**^ and local transformation of HAP (red) to “pseudo-HAP” (orange). Restriction to OH^**−**^ positions in HAP (x, red) and water molecules in “pseudo-HAP” (o, green) as well as H_2_O–molecules (**∙**, green) within the water–rich layers in OCP. Projections along *c* (HAP) and c* (OCP), respectively. (**d**) The HAP-OCP interface model proposed by Fernandez *et al*. where [−12–10] HAP coincide with [010] OCP and where the *c*-HAP-axis is parallel to the *c*-OCP axis. The angle between *a-*HAP and *a*-OCP amounts to 131°.

The influence of osteocalcin on the mineralization steps is of special interest. Besides the monitoring contributions of the type I collagen, osteocalcin takes on the functions of Ca ion transport and suppression of the HAP expansion. The tropocollagen fibrils mineralized by HAP are decorated by individual osteocalcin (OC) molecules, which preferably form pearl necklace strings (Figs. 9e,f). Additionally, OC is perfectly is attached to the (100) planes of HAP and additionally induces epitaxial growth of OCP on HAP (Figs. 9e,f). The Ca^2+^ positions (distances Ca∙∙∙Ca around 9.5 Å) at the (100) faces of HAP and OCP provide an epitactic interface as demonstrated in Figs. 10a,b. Furthermore, the interaction of OC with the (100) planes of HAP and OCP is supported by the fact that the OH groups of HAP can easily react with the protons of the more acidic medium to H_2_O, and by the presence of layers of water molecules, a structure which is characteristic for OCP (Fig. 10c)^13,16,17^. In general, the diffusion (transport) of OC in bone depends on an aqueous environment, which in fact, is realized in the system under investigation on all kinds of interfaces. Fernandez *et al*. proposed another model of HAP-OCP interface, which shows an angle of 131° (Fig. 10d)^24^. Similar relationship could be observed in this work *e*.*g*. in the case of FIB-cut tibia of rat (Fig. 4d).

In the case of the bone structure, obviously osteocalcin assemblies act as confining and structure directing units. Osteocalcin pearl necklaces act as nucleation sites for OCP along the collagen fibrils. Furthermore, in collagen free space osteocalcin leads to the formation of larger OCP crystals, see Figs. 6e–h and *in vitro* results, Fig. 7. Water plays a decisive role in mineralized tissues and structures^27,84,85^. In addition, other NCPs are known to change their structure near calcium ions adopting a globular assembled state. Consequently, being adequately preserved, one cannot rule out multiple candidates such as *e*.*g*. phosphorylated proteins. Thus, in future systematic *in vitro* investigations on the influence of NCPs such as osteopontin, bone sialoprotein *etc*. are needed in order to identify their role in bone formation as reviewed *e*.*g*. by George and Veis^86^. In our former investigations, phosphoserine proved a convenient compound for the modification of calcium phosphate bone cement - collagen composites^56^. For an unequivocal identification of a particular protein in a crowded biochemical milieu such as bone, immunolabeling is highly advantageous^87,88^. As shown by Chen *et al*. on immunolocalized OC stained with uranyl acetate, the arrangement of OC in chain like structures occurs as well as statistic distribution is observed^16^. In addition, our *in vitro* experiments on the influence of osteocalcin on the mineralization of collagen type I show the formation of OCP plates and globular protein structures on the platelet surface similar to structures as observed for bone in this work^31^.

At first sight, the nanoparticular globular structures could represent an inorganic precursor or likewise a pre-impregnated biopolymer such a polymer-induced liquid-precursor as discussed by Olszta or Nudelman^80,81^. Being amorphous, in TEM these biopolymer-precursors are hard to detect. In case of an assumed inorganic crystalline precursor, crystal facets and pronounced lower indexed lattice planes should appear within the spherical structures. Also, due to possible different orientation of the nanoprecursor with respect to the HAP matrix, diffraction contrast should be present, however, such indications could not be proved by TEM.

A recent work (2018) on bones from single and double genetic knockout mice shows that osteocalcin and osteopontin direct mineral growth and thus determine the quality of bone. Especially, it is proposed that osteocalcin and osteopontin regulate bone mineral crystal size and organization, and that they independently determine the crystal shape. OC is found being dominant in the regulation of the physical properties of the bone mineral, whereas osteopontin seems to regulate the mineral composition^89^. Additionally, the scenario developed in this work concerning the decisive role of acidic carboxylic groups preventing apatite growth along (100) plane goes in-line with a proposed model of the Schmidt-Rohr group from 2010 where citric acid having three carboxylic functions is made responsible for the same effect. The authors revealed that the apatite surfaces with strongly bound citrate molecules are preventing crystal growth to the thickness of about 3 nm. By NMR, they showed that bound citrate accounts for 5.5 wt.-% of the organic matter in bone and covers apatite at a density of about one molecule per 2 nm^2^, with its three carboxylate groups at distances of 0.3 to 0.45 nm from the apatite surface^90^.

The OCP crystals occurring in our bone samples are mostly isolated nanocrystals with different orientations encompassing only few unit cells stabilized by osteocalcin. Thus, the OCP crystals are too few and too small to be observed by electron diffraction, which records reflections within the selected area aperture with diameter of several micrometers. Since HAP is the majority phase, the OCP crystal reflections appear also depressed and that is probably the reason why OCP could not be observed yet by other methods such as (micro) Raman spectroscopy, IR, XRD or electron diffraction. An additional problem in diffraction is the detection of the small angle (100) reflection (18.6 Å), which is the clearest evidence of the occurrence of OCP, because this reflection is hidden in the primary beam (Fig. S6c, inset). High–resolution TEM is the only local method besides assumably NMR, which is able to reveal the isolated nanocrystalline OCP. Our results fit well to recent findings published in 2016 by the Weiner group. They detected octacalcium phosphate-like phases in bone based on Raman and X-ray measurements^91^.

## Conclusion

TEM investigations reveal bone ultrastructure formation of human and rat trabeculae at atomic resolution. The collagen triple–helices and microfibrils are mineralized by hydroxyapatite forming a chemical organic-inorganic composite. Individual alpha–chains within the triple–helix are visualized and the typical helical winding periodicities of 0.86, 2.86 and 8.6 nm along the collagen fibril could be resolved. Further analysis of the TEM micrographs reveals that the non–collagenous protein osteocalcin is present at/on the calcified collagen together with octacalcium phosphate. In the Fourier transform of the high-resolution image the fiber-like pattern of (002) HAP reflection is assigned to the mineralized triple–helix whereas the circumferential pattern in the wide angle region of *e*.*g*. prominent (102) reflection corresponds to osteocalcin mineralization. In overview TEM images, the soluble non–collagenous protein osteocalcin is visible as white globular structures of about 2 nm in diameter along the collagen fibrils. The molecular weight of osteocalcin is ~5.5 kDa containing 46–50 amino acids, which in comparison is significantly smaller than of other NCPs such as osteopontin (glycoprotein) with a molecular weight of 44 kDa (33 kDa nascent). Therefore, it can act as favored mediator molecule for the nucleation and growth of HAP as well as OCP in the intrafibrillar space of collagen fibrils. In the case of the absence of the collagen substrate OC promotes the formation of pure OCP platelets. Mostly, osteocalcin appear as single spherical specimen as well as pearl necklace strings attached to the collagen fibrils. It interacts with the calcium sites on the (100) dominated HAP platelets where the binding sites of the globular osteocalcin of 5.45 and 9.5 Å could be visualised.

## Electronic supplementary material

Supporting_information

## Data Availability

The datasets generated during and/or analyzed during the current study are available from the corresponding author on reasonable request.
