# Supplementary material for: First evidence of octacalcium phosphate@osteocalcin nanocomplex as skeletal bone component directing collagen triple–helix nanofibril mineralization
Source: Sci Rep. 2018 Sep 12;8:13696. doi: 10.1038/s41598-018-31983-5 (PMC6135843; doi:10.1038/s41598-018-31983-5)
Supplement: Supplementary file 1 — Supporting_information [file 41598_2018_31983_MOESM1_ESM.pdf]

Supporting information

First evidence of octacalcium phosphate@osteocalcin nanocomplex as skeletal bone component directing collagen triple-helix nanofibril mineralization

Paul Simon,[a*] Daniel Grüner,[b] Hartmut Worch,[c] Wolfgang Pompe,[c] Hannes Lichte,[d] Thaqif El Khassawna,[e] Christian Heiss,[e,f] Sabine Wenisch,[e,g] Ruediger Kniep[a]

[a]*Max-Planck-Institut für Chemische Physik fester Stoffe, Nöthnitzer Str. 40, 01187 Dresden, Germany*

[b]*Forschungszentrum Jülich, Institut für Energie- und Klimaforschung, IEK-2, 52425 Jülich, Germany*

[c]*Institute of Materials Science, Technical University of Dresden, Helmholtzstr. 7, 01069 Dresden, Germany*

[d]*Institute of Structure Physics, Technical University of Dresden, Zum Triebenberg 50, 01328 Dresden Zaschendorf, Germany*

[e] *Institute for Experimental Trauma Surgery, Faculty of Medicine, Justus-Liebig University of Giessen, Germany*

[f] *Department of Trauma, Hand and Reconstructive Surgery, University Hospital of Giessen-Marburg, Giessen, Germany*

[g] *Department of Veterinary Anatomy, Justus-Liebig University of Giessen, Giessen, Germany*

[*] Corresponding author, email: Paul.Simon@cpfs.mpg.de; Phone: +49-351-4646 4227; Fax: +49-351-4646 4002

1. FIB preparation:	Imaging of collagen fibrils and discussion of preparation artefacts.
2. Beam damage:	TEM experiments and discussion.
3. Figure S1	Light microscopy of trabecular rat tibia (sample for FIB cut).
4. Figure S2	SEM of trabecular rat tibia (sample for FIB cut).
5. Figure S3	SEM of trabecular rat tibia (sample for FIB cut).
6. Figure S4	SEM of trabecular rat tibia (sample for FIB cut).
7. Figure S5	SEM of trabecular rat tibia (sample for FIB cut).
8. Figure S6	SEM and TEM of trabecular rat tibia (sample for FIB cut).
9. Figure S7	SEM and semithin section of human cancellous bone (femur), (diamond).
10. Figure S8	TEM of rat vertebra, overview TEM (diamond cut).
11. Figure S9	Beam damage experiments (sample for FIB cut): Time dependent high–resolution at dose rate and magnification as used for the actual experiment.
12. Figure S10	Beam damage experiments (sample for FIB cut): Time dependent high–resolution at high dose rate and high magnification.
13. Figure S11	Beam damage experiments (FIB cut of biomimetic sample): Imaging triple–helical fibrils at high–resolution in fluorapatite –gelatine composites.

1. FIB preparation: Imaging of collagen fibrils and discussion of preparation artefacts

The combination of focused ion beam (FIB) TEM sample preparation and transmission electron microscopy offers several advantages for submicron and nanostructural investigations. The FIB technique involves specimen thinning using a beam of ions of sufficient energy to remove material. There are several advantages of this technique compared to the usually used microtomy, one benefit is eliminating the need for time–consuming, multi–stage chemical fixation and embedding procedure. Additionally, the mechanical sectioning with a diamond knife potentially can introduce mechanical damages of such brittle objects. As disadvantage it should be mentioned that artifacts are created by the high energy gallium beam such as destruction of the inorganic crystal structure (glass formation) and damaging of the organic components. Alteration of FIB samples surfaces due the irradiation with gallium ions cannot be avoided and causes artifacts such as redeposition of sputtered material, Ga ion implantation and surface amorphization.[1] Beam damage emerges as beam–induced heating, knock–on damage and radiolysis. Beam–induced heating occurs due to the generation of phonons and depends on the ion beam current, the ion voltage and the thermal conductivity of the sample. Biological materials typically have 100 times lower thermal conductivities compared to metals or semiconductors which may lead in worst case to melting. Knock–on damage, in which gallium ions physically displace sample atoms, produces a heavily disordered or amorphous surface damage layer.[1,2] The knock–on damage can be directly measured in crystalline samples by determining the width of the amorphous zone at the edge of the sample using high–resolution TEM imaging.[3] Detectable alteration of the surface chemistry could also occur, as knock-on displacements may preferentially remove complete functional groups as a single unit, including entire side groups on long bio–polymer chains. Radiolysis damage involves irreversible changes in the electronic structure of a material due to incoming ionizing radiation, which can include the destruction of chemical bonds and changes in chemical coordination. Radiolysis in samples can occur from both the electron beam and the charged ion beam. Radiolysis damage in biological materials may result in a change of appearance and the preferential destruction or creation of organic functional groups. Nevertheless, it is possible to produce FIB samples of high quality for even atomic resolution studies. In our case deposited platinum coating with thickness in the

range of tens of nanometers prevented the charging of the sample. Another critical feature of FIB is the small field of view of about 10–30 μm, which only allows a targeted preparation of area of interest.

Often, the FIB technique was used and proved to be successful to prepare TEM specimens of artificial bone–like or bioinspired systems such as of hydroxyapatite thin films grown on metallic substrates or fluorapatite–gelatine composites, see Iliescu *et al.* and Simon *et al* in 2004.[4,5] TEM (EELS) examinations of natural systems such as enamel or dentine prepared by FIB have been reported *e.g.* by Hoshi *et al*. already in 2001.[6] Further on, in 2004 Engquist *et al.* imaged the interface of enamel and a bioactive ceramic repair material and in 2005, Nalla *et al.* imaged collagen fibrils in dentine.[7,8] Further FIB work on bone was performed by Porter *et al.* in 2005 where the morphology and surface between bone and bone ceramics was characterized.[9] Mostly, FIB was used to generate polished surfaces in order to carry out EDX investigations on the bone/implant or the cell/bone-ceramic interface.[10,11] In 2007, Giannuzzi *et al.* performed 3D scanning electron microscopy analysis of bone/dental implant interfaces and showed high–resolution images of hydroxyapatite crystals of bone of FIB prepared samples.[12] In 2008, Palmquist *et al.* published high–resolution images of HAP at the bone-titanium implant interface of human forearm bone–anchored amputation prosthesis.[13] This group mostly characterized the interface morphology of implants and bone by using EFTEM and high–resolution TEM[14-21] extended to electron tomography on bone–titanium oxide interface in humans at the nanometer scale.[19,22] In the above mentioned cases, it was proved by TEM imaging that the collagen fibril is not affected and that the 67 nm striation is preserved by the FIB milling procedure.[8,17] Besides gallium ion milling also argon ion beam polishing is used which is a fast preparation technique for evaluating interfaces of osseo–integrated implants.[23] In the course of our former extensive investigations we could show that in case of the beam sensitive organic/inorganic composites prepared by FIB milling encompassing human and artificial otoconia, bovine teeth, oxalate dihydrate polyacrylic acid composites, desmosponges, layered organic solar cells, nanocolloidal mesocrystals and gelatin–fluorapatite composites the sample structure is preserved.[5,23-32] For example, high–resolution of the apatite (002) lattice of 3.44 Å combined with the imaging of individual collagen triple–helices proved to be possible in FIB cut samples of fluorapatite–

gelatine composites.[5,31] FIB cut samples prepared in cross section can be helpful to image repair layers covering pain sensitive nerve channels of bovine dentin at high–resolution. The dentin repair was achieved by applying specific toothpaste containing a composite of nanosized hydroxyapatite and gelatine.[32]

In our study we used FIB cuts of rat tibia (see Figure 1,2,4,5,S6,S9,S10 and SEM images S1-S4). In order to rule out artefacts caused by the FIB cut preparation we also investigated conventional diamond thin cuts of human femur (Figure 3,8,9 and Figure S7) and rat vertebra (Figure 6 and Figure S7).

2. Beam damage in TEM: Experiments and discussion

In order to test and to determine the stability of the FIB cut sample, we performed time–dependent electron irradiation experiments at harsh high–resolution conditions (Figure S10a–c) of the area presented in Figure 5. The applied electron dose rate amounted about 2000 electrons per square Angström and per second which is double of the dose rate used in Figure S9b,c and much more accumulated dose which exceeded ~25 (Figure S10b) and ~110 times (Figure S10c) the actual applied dose (Figure 5b). After several recording and focusing sequences, the crystal structure remained as indicated by the corresponding Fourier transforms of the high–resolution images (Figure S10a-c, bottom). Only after three minutes persistent radiation and about 493.500 e/Å^2 accumulated dose the crystal showed changes under irradiation, see Figure S10c, arrow, whereas the FFT seems still not to be affected, keeping the resolution of 1.48 Å. Therefore, for this area we exclude that under the actual recording conditions the crystal is already affected. In order to prove that the observed structures are not simply beam damage artifacts, we recorded high–resolution images with high electron doses. In this way, we take into account damage that is caused by the additional time needed for focusing the sample and for recording the images at high magnification. As we have already shown in numerous publications the triple–helical structure is well preserved in the apatite structure in FIB cuts[5,31] as well in classical diamond ultramicrotomy.[33,34] In Figure S11a,b high–resolution is shown from a FIB cut of a fluorapatite–gelatine composite, where a bundle of triple–helices is revealed. Generally, formation of bubbles

is observed during decomposition of bulk apatite samples due to electron irradiation with a high dose in the TEM[35] and also formation of CaO.[36,37] In our case, formation of bubbles and CaO was not observed. Application of higher electron dose even may lead to increased crystallinity in bone as reported by Hong *et al.*[38] or transition of hexagonal to monoclinic apatite.[39] In case of OCP the beam damage should be a higher concern than in apatite because of the presence of hydrated layers. Nevertheless, as shown by *e.g.* Suvorova and Buffat using a field emission microscope at 300 kV and magnification as high as 510.000x to 640.000x it is possible to image OCP in high–resolution.[40] Xin *et al.* also imaged successfully OCP in high–resolution at 200 kV with field emission and tested the stability of OCP by different recording times.[41] Also, our group investigated thin, biomimetically grown OCP platelets in high–resolution which were synthesized to be used as dentine repair material.[32] Bres *et al.* applied high doses needed for STEM by EELS. They state that dose rates of 160 A/cm^2 corresponding to nearly 100.000 e/Å^2 and even doses of 350-2500 A/cm^2 do not cause remarkable beam damage in HAP.[36]

When one performs electron microscopy on organic and biological materials, beam damage will play a decisive role.[42-48] Therefore, the attainable resolution is not given by the point resolution of the microscope but by the critical electron dose on the sample. Various undesirable phenomena will occur, such as loss of high–resolution structural information as well as long–range order accompanied by chemical decomposition, mass loss, and decrease of mechanical stability. Finally, with the higher brightness of the field emission guns (FEG) and higher acceleration voltage, the probability of knock–on damage will also increase for organic and biological samples. Therefore, it is important to estimate roughly the influence of the electron beam damage on unstained biological samples. In general, proteins are extremely beam sensitive, and some are even destroyed by a dose of ~1 e Å^{-2}.[42] Former measurements by electron diffraction on glucose embedded bacteriorhodopsin[47] show that the finer structural detail of 7 Å vanishes at a dose of 1.4 e Å^{-2}, and that the 3 Å reflection already reaches the critical dose below 1 e Å^{-2} at room temperature. In a very early paper by Glaeser *et al.* the beam damage of glucose embedded catalase crystals is described.[48] They found that the logarithm of critical electron dose is linear with the achievable resolution. In summary, for biological samples, the main restriction of resolution is given by the beam sensitivity of the protein and thus only a low dose of about 1–

10 e Å^{-2} can be applied. For example, if a dose of 10 e Å^{-2} is exerted, one should be able to measure ~3 nm as smallest object detail. Cryo TEM studies are achieving reproducible 3D resolutions in the order of 6 to 10 Å as shown on an archeon S–layer displayed structural details of ~10 Å at –120 °C with spot scan illumination using a total dose of about 1 e Å^{-2}.[49] Kimura *et al*. imaged the surface of bacteriorhodopsin with a resolution of ~4 Å at 4.2 K, using low dose and an acceleration voltage of 400 kV.[50] The total dose amounted to about less than 10 e Å^{-2}. Thus, by using a cooling holder, with low dose and by embedding the sample into a polymer matrix combined with higher acceleration voltage, one is be able to reach at best a resolution of 4 Å. Another possibility is to use reduced acceleration voltage of 80 kV since the knock–on damage threshold of carbon atoms lies above this range.[51] Nowadays promising steps are taken in the direction of even applying 20 kV by using chromatic aberration correction for atomic resolution combined with spherical aberration–corrector.[52]

In our actual experiments, beam damage artefacts should be minimized, since we used the low-dose technique, meaning focusing on a vicinal area and using low illumination. Also, we applied lower magnification of about 320.000x than usual (500.000–800.000x). In addition, collagen is a highly interconnected biopolymer and thus more stable than the above discussed proteins. For example, in our former holography experiments we used 24 e Å^{-2} at 200 kV for imaging the 67 nm striation pattern without occurrence of beam damage of a non–stained collagen sample.[53] In another work, we were able to record HR–TEM images of silicification of collagen on *H. sieboldii* glass sponges.[54] Finally, we experienced since over a decade of TEM and FIB cut preparation on apatite–gelatine composites that the apatite serves as a protection matrix and stabiliser for the buried and integrated protein inside.[5,31,32]

References for supporting information

1. Giannuzzi, L. A.; Stevie, F. A. *Introduction to focused ion beams: instrumentation, theory, techniques and practice.* Springer: New York, 2005.

2. McCaffrey, J. P.; Phaneuf, M. W.; Madsen, L. D. Surface Damage Formation during Ion-Beam Thinning of Samples for Transmission Electron Microscopy. *Ultramicroscopy* **2001**, *87*, 97-104.

3. Mayer, J.; Giannuzzi, L. A.; Kamino, T.; Michael, J. TEM Sample Preparation and FIB-Induced Damage. *MRS Bull*. **2007**, *32*, 400-407.

4. Iliescu M.; Nelea V.; Werckmann J.; Mihailescu, I. N. Transmission Electron Microscopy Investigation of Pulsed-Laser Deposited Hydroxylapatite Thin Films Prepared by Tripod and Focused Ion Beam Techniques. *Surf. Coat. Technol*. **2004**, *187*, 131-140.

5. Simon, P.; Carrillo-Cabrera, W.; Formanek, P.; Göbel, C.; Geiger, D.; Ramlau, R.; Tlatlik, H.; Buder, J.; Kniep. R. On the Real-Structure of Biomimetically Grown Hexagonal Prismatic Seeds of Fluorapatite-Gelatine-Composites: TEM Investigations along [001]. *J. Mater. Chem.* **2004**, *14*, 2218-2224.

6. Hoshi K.; Ejiri S.; Probst W.; Seybold, V.; Kamino, T.; Yaguchi, T.; Yamahira, N.; Ozawa, H. Observation of Human Dentine by Focused Ion Beam and Energy-Filtering Transmission Electron Microscopy. *J. Microsc. Oxford* **2001**, *201*, 44-49.

7. Engqvist, H.; Schultz-Walz, J. E.; Loof, J.; Botton, G. A.; Mayer, D.; Phaneuf, M. W.; Ahnfelt, N. O.; Hermansson, L. Chemical and Biological Integration of a Mouldable Bioactive Ceramic Material Capable of Forming Apatite in Vivo In Teeth. *Biomaterials* **2004**, *25*, 2781-2787.

8. Nalla R. K.; Porter A. E. Daraio C. Minor, A. M.; Radmilovic, V.; Stach, E. A.; Tomsia, A. P.; Ritchie, R. O. Ultrastructural Examination of Dentin Using Focused Ion-Beam Cross-Sectioning and Transmission Electron Microscopy. *Micron* **2005**, *36*, 672-680.

9. Porter A. E.; Buckland T.; Hing K.; Best, S. M.; Bonfield, W. The Structure of the Bond Between Silicon-Substituted Hydroxyapatite Bone and Porous Bioceramic Implants. *J. Biomed. Mater. Res. Part A*, **2006**, *78A*, 25-33.

10. Engqvist H; Botton G. A.; Couillard M.; Mohammadi, S.; Malmstrom, J.; Emanuelsson, L.; Hermansson, L.; Phaneuf, M. W.; Thomsen, P. A Novel Tool for High-Resolution Transmission Electron Microscopy of Intact Interfaces Between Bone and Metallic Implants. *J. Biomed. Mater. Res. Part A*, **2006**, *78A*, 20-24.

11. Edwards H. K.; Fay M. W.; Anderson S. I.; Scotchford, C. A.; Grant, D. M.; Brown, P. D. An Appraisal of Ultramicrotomy, FIBSEM and Cryogenic FIBSEM Techniques for the Sectioning of Biological Cells on Titanium Substrates for TEM Investigation. *J. Micr.* **2009**, *234*, 16-25.

12. Giannuzzi L. A.; Phifer D.; Giannuzzi N. J.; Capuano, M. J. Two-Dimensional and 3-Dimensional Analysis of Bone/Dental Implant Interfaces with the Use of Focused Ion Beam and Electron Microscopy. *J. Or. Maxillofac. Surg,* **2007**, *65*, 737-747.

13. Palmquist A; Jarmar T.; Emanuelsson L; Branemark, R.; Engqvist, H.; Thomsen, P. Forearm Bone-Anchored Amputation Prosthesis - A Case Study on the Osseointegration. *Act. Orth.,* **2008**, *79*, 78-85.

14. Palmquist A.; Jarmar T.; Hermansson L.; Emanuelsson, L.; Taylor, A.; Taylor, M.; Engqvist, H.; Thomsen, P. Calcium Aluminate Coated and Uncoated Free Form Fabricated CoCr Implants: A Comparative Study in Rabbit. *J. Biomed. Mater. Res. Part B - Appl. Biomater.*, **2009**, *91B*, 122-127.

15. Palmquist A.; Lindberg F.; Emanuelsson L.; Branemark, R.; Engqvist, H.; Thomsen, P. Morphological Studies on Machined Implants of Commercially Pure Titanium and Titanium Alloy (Ti6Al4V) in the Rabbit. *J. Biomed. Mater. Res. Part B Appl. Biomater*. **2009**, *91B*, 309-319.

16. Palmquist A.; Lindberg F.; Emanuelsson L.; Branemark, R.; Engqvist, H.; Thomsen, P. Biomechanical, Histological, and Ultrastructural Analyses of Laser Micro- and Nano-Structured Titanium Alloy Implants: A Study in Rabbit. *J. Biomed. Mater. Res. Part A* **2009**, *92A*, 1476-1486.

17. Palmquist A.; Omar O. M.; Esposito M.; Lausmaa, J.; Thomsen, P. Titanium Oral Implants: Surface Characteristics, Interface Biology and Clinical Outcome. *J. Soc. Interf*. **2010**, *7*, S515-S527.

18. Grandfield, K.; McNally, E. A.; Palmquist, A.; Botton, G. A.; Thomsen P.; Engqvist. H. Visualizing Biointerfaces in Three Dimensions: Electron Tomography of the Bone - Hydroxyapatite Interface. *J. R. Soc. Interf.* **2010,** *7*, 1497-1501.

19. Grandfield K.; Palmquist A.; Goncalves S.; Taylor, A.; Taylor, M.; Emanuelsson, L.; Thomsen, P.; Engqvist, H. Free Form Fabricated Features on CoCr Implants with and without Hydroxyapatite Coating in Vivo: A Comparative Study of Bone Contact and Bone Growth Induction. *J. Mater. Sci. Mater. Med*. **2011**, *22*, 899-906.

20. Palmquist A.; Emanuelsson L.; Branemark R.; Thomsen, P. Biomechanical, Histological and Ultrastructural Analyses of Laser Micro- and Nano-Structured Titanium Implant after 6 Months in Rabbit. *J. Biomed. Mater. Res. B Appl. Biomater.* **2011**, *97B*, 289-298.

21. Omar O. M.; Lenneras M. E.; Suska F.; Emanuelsson, L.; Hall, J. M.; Palmquist, A.; Thomsen, P. The Correlation between Gene Expression of Proinflammatory Markers and Bone Formation during Osseointegration with Titanium Implants. *Biomaterials* **2011**, *32,* 374-386.

22. Palmquist A.; Grandfield K.; Norlindh B.; Mattsson, T.; Branemark, R.; Thomsen, P. Bone-Titanium Oxide Interface in Humans Revealed by Transmission Electron Microscopy and Electron Tomography. *J. Roy. Soc Interf.* **2012**, *9*, 396-400.

23. Grüner D.; Faldt J.; Jansson K.; Shen, Z. J. Argon Ion Beam Polishing: A Preparation Technique for Evaluating the Interface of Osseointegrated Implants with High Resolution. *Int. J. Or. Maxillofac. Implants,* **2011**, *26,* 547-552.

24. Ehrlich, H.; Simon, P.; Carrillo-Cabrera, W.; Bazhenov, V. V.; Botting, J. P. ; Ilan, M.; Ereskovsky, A. V.; Muricy, G.; Worch, H.; Mensch, A. *et al.* Insights into Chemistry of Biological Materials: Newly Discovered Silica-Aragonite-Chitin Biocomposites in Demosponges. *Chem. Mater*. **2010**, *22*, 1462-1471.

25. Simon, P.; Rosseeva, E.; Baburin, I. A.; Liebscher, L.; Hickey, S. G.; Cardoso-Gil, R.; Eychmüller, A.; Kniep, R.; Carrillo-Cabrera, W. Relationship between Atomistic Arrangement and Internal Nanocrystal Super- Structure Ordering within Pbs-Organic Colloidal Nanocrystals *Ang. Chem. Int. Ed.* **2012,** *51*, 10776 –10781.

26. Thomas, A.; Rosseeva, E.; Hochrein, O.; Carrillo-Cabrera, W.; Simon, P.; Duchstein, P.; Zahn, D.; Kniep, R. Mimicking the Growth of a Pathologic Biomineral: Shape Development and

Structures of Calcium Oxalate Dihydrate in the Presence of Polyacrylic Acid. *Chem. Eur. J.* **2012**, *18,* 4000-4009.

27. Simon, P.; Carrillo-Cabrera, W.; Huang, Y.-H.; Buder, J.; Borrmann, H.; Cardoso-Gil, R.; Rosseeva, E.; Yarin, Yu.; Zahnert, T.; R. Kniep, R. Structural Relations Between Calcite-Gelatine Composites and Biogenic (Human) Otoconia. *Eur. J. Inorg. Chem.* **2011,** 5370-5377.

28**.** Huang, Y.-X.; Buder, J.; Cardoso, R.; Prots, Y.; Carrillo-Cabrera, W.; Simon, P.; Kniep, R. Shape Development and Structure of a Complex (Otoconia-Like?) Calcite-Gelatine Composite. *Ang. Chem. Int. Ed.* **2008,** *47*, 8280-8284.

29. Rosseeva, E.; Buder, J.; Simon, P.; Kniep, R. Synthesis, Characterization, and Morphogenesis of Carbonated Fluorapatite-Gelatine Nanocomposites: A Complex Biomimetic Approach toward the Mineralization of Hard Tissues. *Chem. Mater.* **2008,** *20*, 6003–6013.

30. Simon, P.; Lichte, H.; Drechsel, J.; Formanek, P.; Graff, A.; Wahl, R.; Mertig, M.; Adhikari, R.; Michler, G. H. Electron Holography of Organic and Biological Materials. *Adv. Mater.* **2003**, *15*, 1475-81.

31. Kniep, R; Simon, P. Hidden" Hierarchy of Microfibrils within 3D-Periodic Fluorapatite–Gelatine Nanocomposites: Development of Complexity and Form in a Biomimetic System. *Angew. Chem. Int. Ed.* **2008**, *47,* 1405 –1409.

32. Kollmann, T.; Simon, P.; Carrillo-Cabrera, W.: Braunbarth, C.; Poth, T.; Rosseeva, E.; Kniep, R. Calcium Phosphate-Gelatin Nanocomposites: Bulk Preparation (Shape- and Phase-Control), Characterization, and Application as Dentine Repair Material. *Chem. Mater.* **2010**, *22*, 5137-5153.

33. Simon, P.; Rosseeva, E.; Buder, J.; Carrillo-Cabrera, W.; R. Kniep, R. Embryonic States of Fluorapatite-Gelatine-Composites and Their Intrinsic Electric Field Driven Morphogenesis: The Missing Link on the Way from Molecular Dynamics Simulations to Pattern Formation on the Meso-Scale. *Adv. Func. Mater.* **2009**, *19,* 3596-3603.

34. Grüner, D.; Lips, K. S.; Heiss, C.; Schnettler, R.; Kollmann, T.; Simon, P.; Kniep, R. Irregular, Possibly Semi-Crystalline Platelet Deposition at the Mineralization Front of Rabbit Femur Osteotomy: A HR-TEM Study. *Scanning* **2013**, *35*, 169-82.

35. Meldrum A.; Wang, L. M.; Ewing R. C. Electron-Irradiation-Induced Phase Segregation in Crystalline and Amorphous Apatite: A TEM study. *Am. Mineral.* **1997**, *82*, 858-869.

36. Brès, É. F.; Reyes-Gasga, J.; Rey, C.; Michel, J. Probe Size Study of Apatite Irradiation in STEM. *Eur. Phys. J. Appl. Phys*. **2014,** *67*, 1-6.

37. Reyes-Gasga, J.; Garcia-Garcia R. Analysis of the Electron-Beam Radiation Damage of TEM Samples in the Acceleration Energy Range From 0.1 to 2 meV Using the Standard Theory for Fast Electrons. *Rad. Phys. Chem.* **2002**, *64*, 359–367.

38. Hong, S. I.; Hong, S. K.; Wallace, J. M.; Kohn D. H. Ultrastructural Observation of Electron Irradiation Damage of Lamellar Bone. *J. Mater. Sci.-Mater. Med*. **2009**, *20*, 959–965.

39. Espanol, M.; Portillo, J.; Manero, J. M.; Ginebra, M. P. Investigation of the Hydroxyapatite Obtained as Hydrolysis Product of Alpha-Tricalcium Phosphate by Transmission Electron Microscopy. *Cryst. Eng. Comm.* **2010**, *12*, 3318–3326.

40. Suvorova, E. I.; Buffat, P.A . Electron Diffraction and High Resolution Transmission Electron Microscopy in the Characterization of Calcium Phosphate Precipitation from Aqueous Solutions under Biomineralization Conditions. *Eur. Cells Mater*. **2001**, *1*, 27.

41. Xin, R.; Leng, Y.; Wang, N. In Situ TEM Examinations of Octacalcium Phosphate to Hydroxyapatite Transformation. *J. Cryst. Growth* **2006**, *289*, 339-344.

42. Knapek, E. Properties of Organic Specimens and their Supports at 4 K under Irradiation in an Electron Microscope. *Ultramicroscopy* **1982**, *10*, 71-86.

43. Workshop on High-Resolution Electron Microscopy, **2000**. Betheseda, MD, USA, http://em-outreach.sdsc.edu/web-course.

44. Grubb, D. T. Radiation Damage of Organic Materials in the Transmission Electron Microscope. *Ultramicroscopy* **1984**, *12*, 279-280.

45. Kellenberger, E. The Ups and Downs of Beam Damage, Contrast and Noise in Biological Electron Microscopy. *Micron Microsc. Act.* **1986**, *17*, 107-114.

46. Brink, J.; Gross, H.; Tittmann, P.; Sherman, M. B.; Chiu, W. Reduction of Charging in Protein Electron Cryomicroscopy. *J. Microsc.* **1998**, *191*, 67-73.

47. Stark, H.; Zemlin, F.; Boettcher, C. Electron Radiation Damage to Protein Crystals of Bacteriorhodopsin at Different Temperatures. *Ultramicroscopy* **1996,** *63*, 75-81.

48. Glaeser, R. M.; Taylor, K. A. Radiation-Damage Relative to Transmission Electron-Microscopy of Biological Specimens at Low-Temperature – Review. *J. Microsc*. **1978**, *112*, 127-138.

49. Cheong, G. W.; Typke, D.; Baumeister, W. Projection Structure of the Surface Layer of Methanoplanus Limicola at 10 Angstrom Resolution Obtained by Electron Cryomicroscopy. *J. Struct. Biol.* **1996**. *117,* 138-144.

50. Kimura, Y.; Vassylyev, D. G.; Miyazawa, A.; Kidera, A.: Matsushima, M.: Mitsuoka, K.; Murata, K.; Hirai, T.; Fujiyoshi, Y. Surface of Bacteriorhodopsin Revealed by High-Resolution Electron Crystallography. *Nature* **1997**, *389*, 206-211.

51. Banhart, F. Irradiation Effects in Carbon Nanostructures. *Rep. Prog. Phys.* **1999,** *62*, 1181–1221.

52. Linck, M.; Hartel, P.; Uhlemann, S.; Kahl, F.; Müller, H.; Zach, J.; Haider, M. Chromatic Aberration Correction for Atomic Resolution TEM Imaging from 20 to 80 kV. *Phys. Rev. Lett.* **2016,** *117,* 076101.

53. Simon, P.; Lichte, H.; Formanek, P.; Lehmann, M.; Huhle, R.; Carrillo-Cabrera, W.; Harscher, A.; Ehrlich, H. Electron Holography of Biological Samples. *Micron* **2008**, *39*, 229–256.

54. Ehrlich, H.; Deutzmann, R.; Brunner, E.; Cappellini, E.; Koon, H.; Solazzo, C.; Yang, Y.; Ashford, D.; Thomas-Oates, J.; Lubeck, M. *et al.* Mineralization of the Metre-Long Biosilica Structures of Glass Sponges is Templated on Hydroxylated Collagen. *Nat. Chem.* **2010**, *2*, 1084-1088.

3. Figure S1

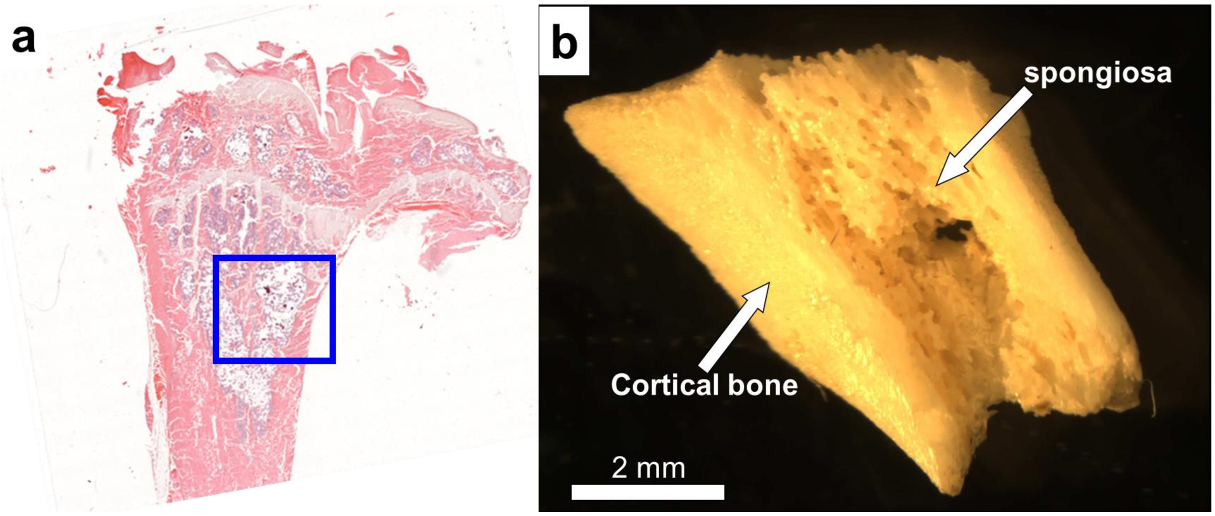

Figure S1. (a) General appearance of the tibia head stained by hematoxylin and eosin. (b) Light microscopy image of the tibia head sample obtained by mechanical destruction corresponding to the blue frame area of (a).

4. Figure S2

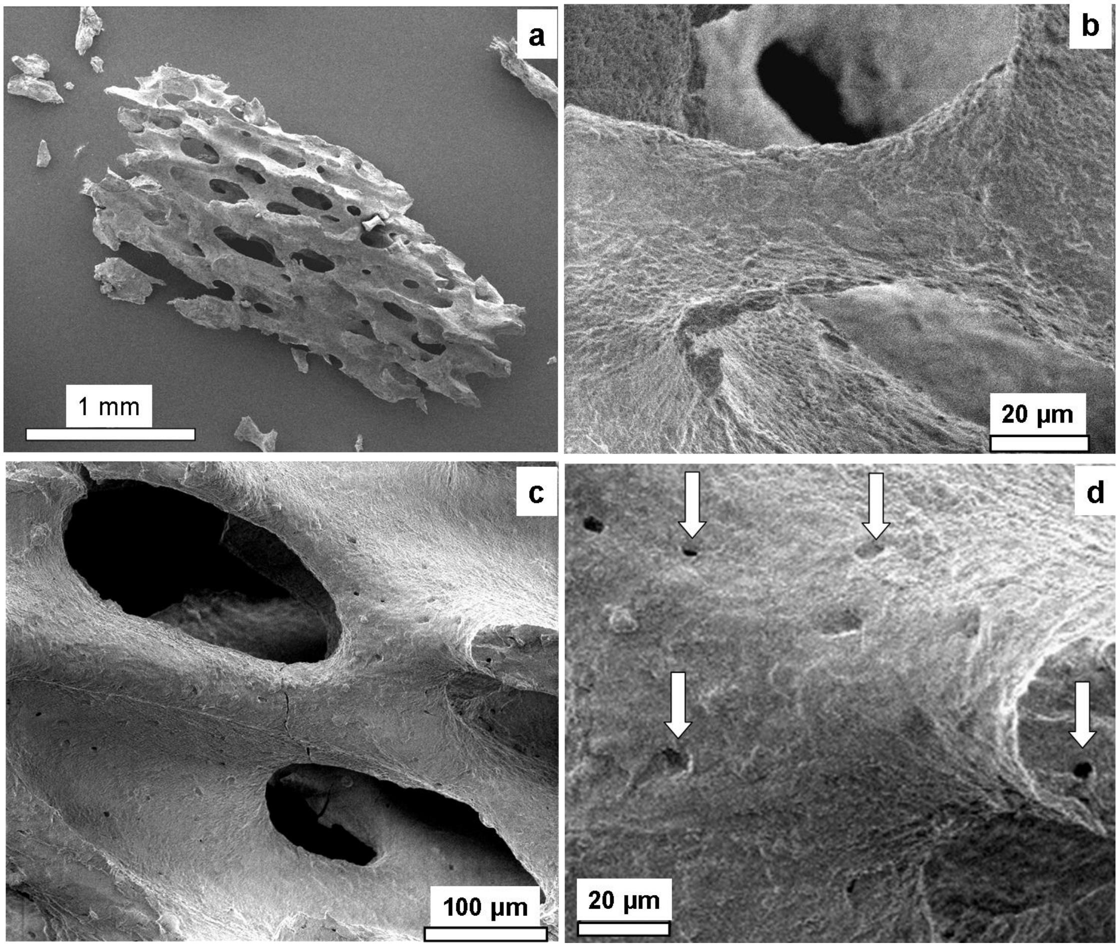

Figure S2. (a) Overview SEM image of a spongy fragment of the bone interior with a size of about (2x1) mm. (b) Rough surface pattern of a single trabecula with assembly of small patches 5–20 μm in size. (c) Spongiosa consists of trabeculae of about 50–100 μm thickness with oval pores between the trabeculae having a maximum size of 200 μm. (d) Smaller pores are patterning the trabecula surface with diameters between 3 and 6 μm (see white arrows).

5. Figure S3

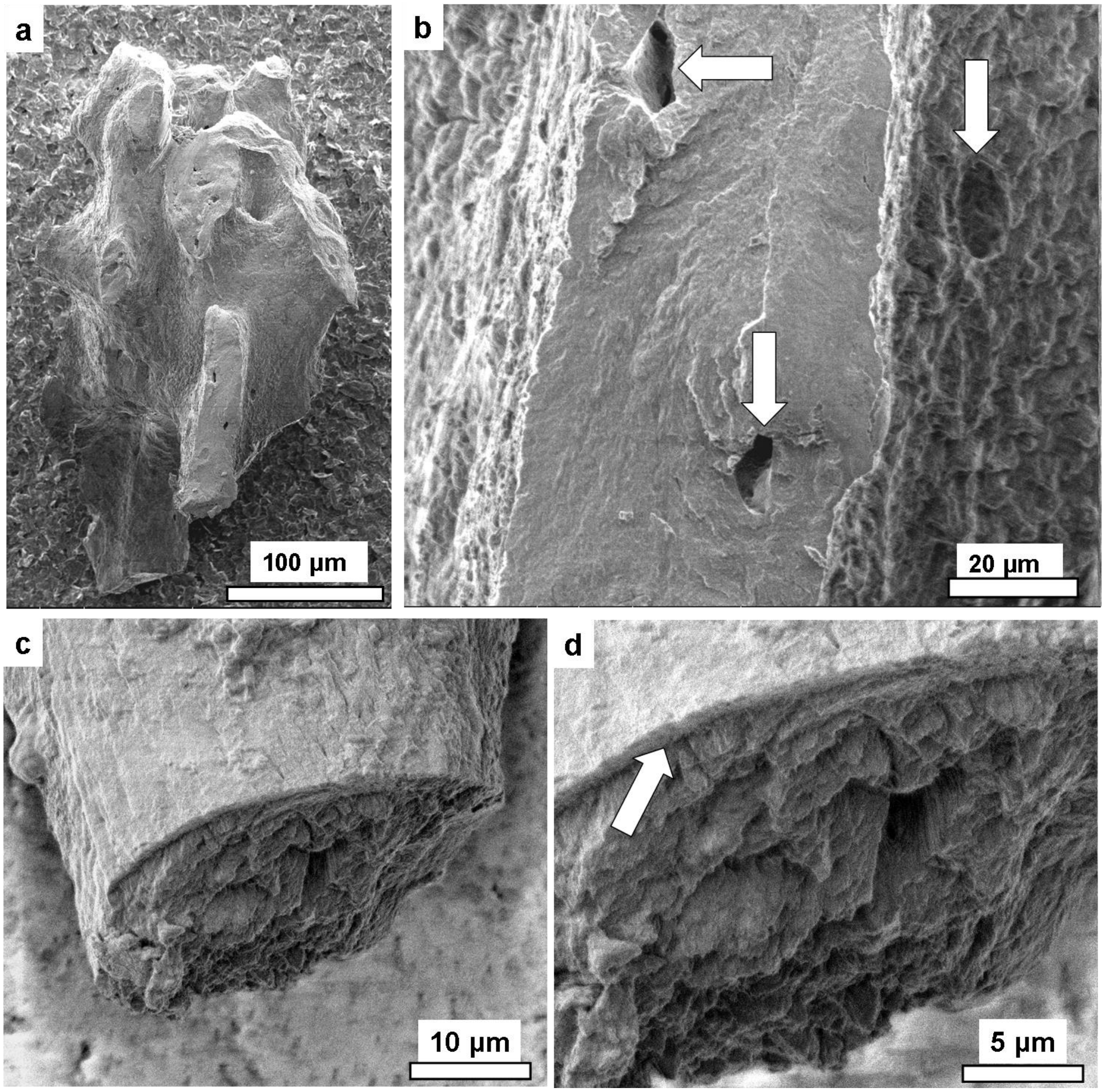

Figure S3. (a) Image displaying a broken spongy piece. Several trabeculae originate from a massive central part. (b) Zoomed image of the fracture surface of a trabecula with tiny holes 5–10 μm in diameter revealing a channel system interspersing the spongy bone. (c,d) Cross-sectional fracture surface of a single trabecula indicating a core/shell motif (shell marked with arrow).

6. Figure S4

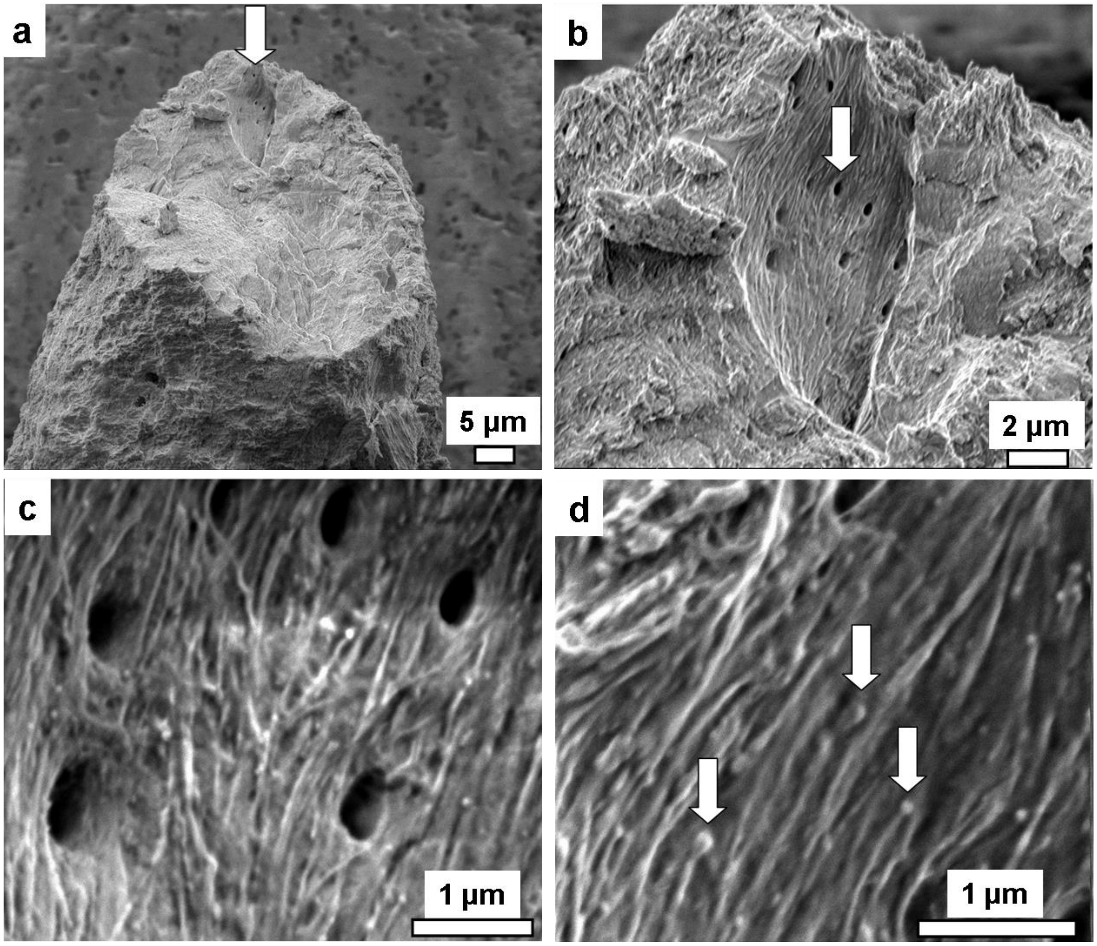

Figure S4. (a) Smooth cleavage surface of a trabecula with a diameter of about 40 μm revealing a pore inside (see arrow). (b) Enlarged view of the hole with 6 μm in diameter (white arrow). (c) The walls of the hole are smooth and show a system of nanochannels with a diameter of about 500 nm. The wall is decorated with fine fibrils with a diameter of about 20 nm, mostly aligned along the trabecula long axis. (d) Nodule shaped endings of the fibrils (see arrows).

7. Figure S5

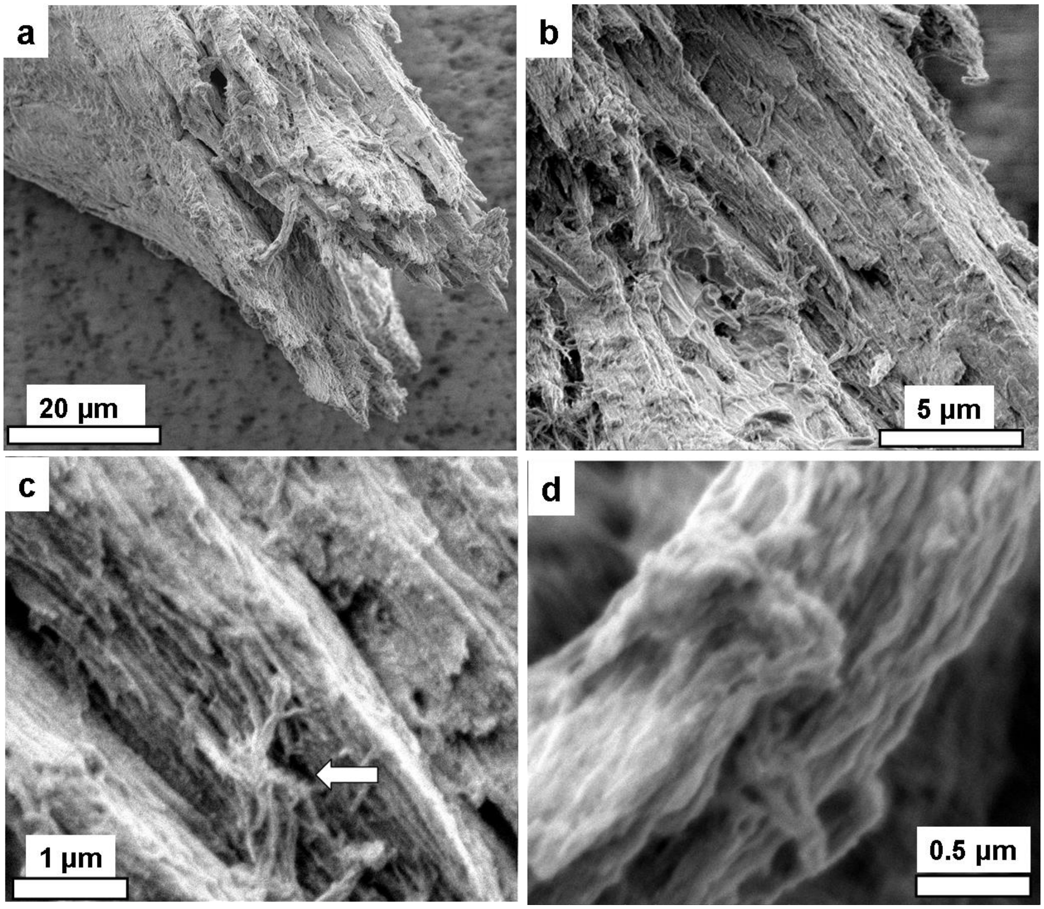

Figure S5. (a) Broken trabecula tip with layered surface structure. The layer thickness is about 1–2 µm suggesting an orthogonal plywood structure. (b) Enlarged view of layered pattern. (c) Each layer is interconnected by bundles of fibrils (white arrow). Layers are divided in sublayers with a thickness of about 100–150 nm. (d) Hierarchical assembly of a micrometer strong fibril with sub–filaments up to 20 nm thickness.

8. Figure S6

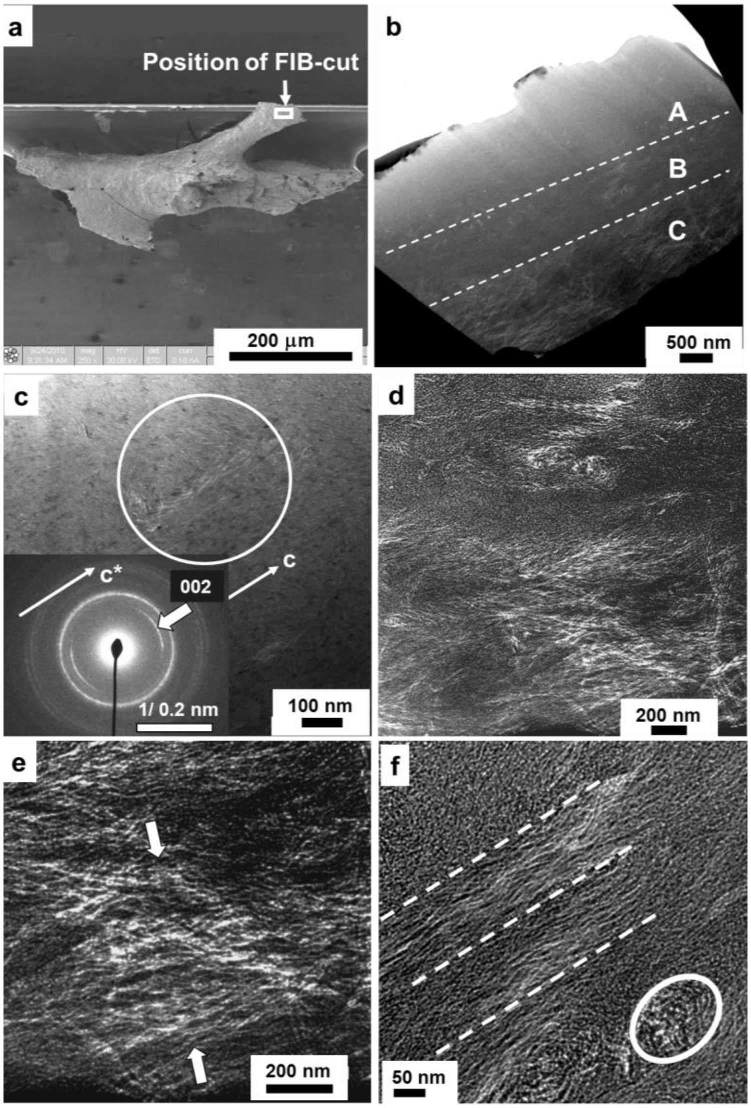

Figure S6. Trabecula of rat: Preparation (a), characterization and TEM investigation of a FIB cut (b–f). (b) Overview: Areas A, B and C with different thicknesses. (c) Enlargement of B: White circle indicates the collagen fibrils selected for electron diffraction. (Inset c) Electron diffraction pattern of the white circle region containing collagen fibrils. (d,e) Collagen fibrils showing typical striation of 67 nm (arrows) imaged from thick area C. (f) Enlarged view showing fibrils in parallel orientation (between broken lines) and along the view direction (bottom ellipsoid).

9. Figure S7

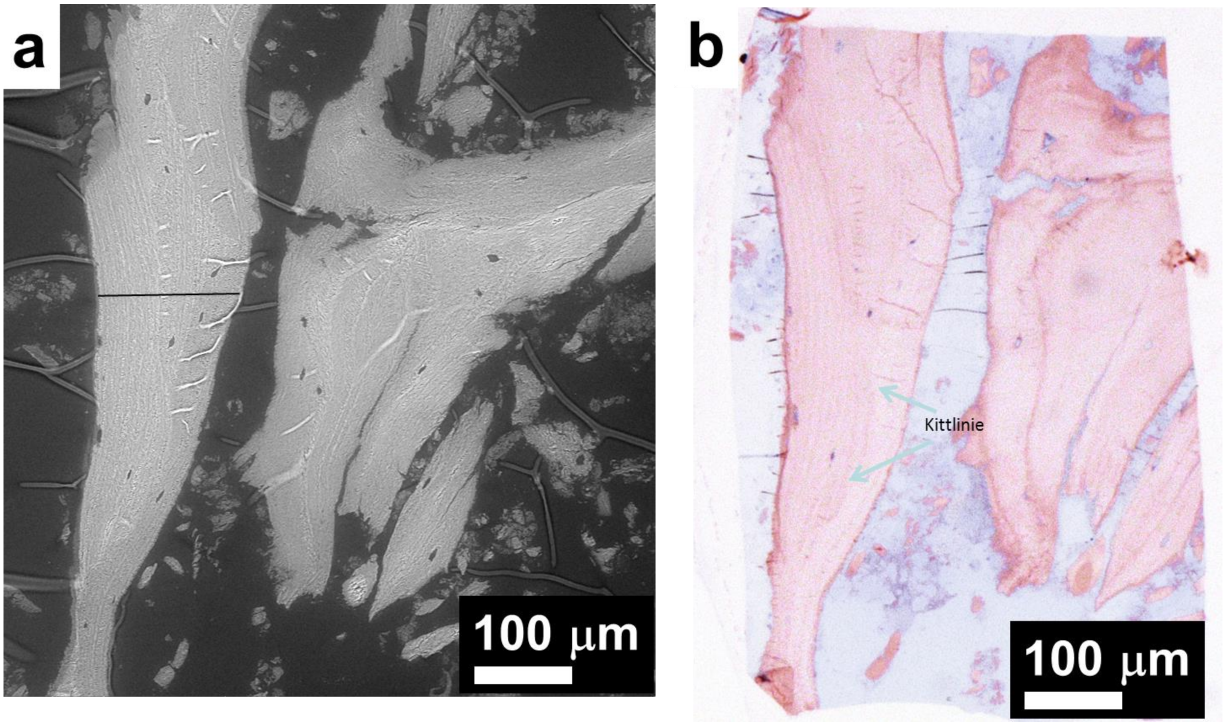

Figure S7. (a) Overview SEM back scattered image of human cancellous bone ultramicrotomy. (b) Stained light microscopic image of same region.

10. Figure S8

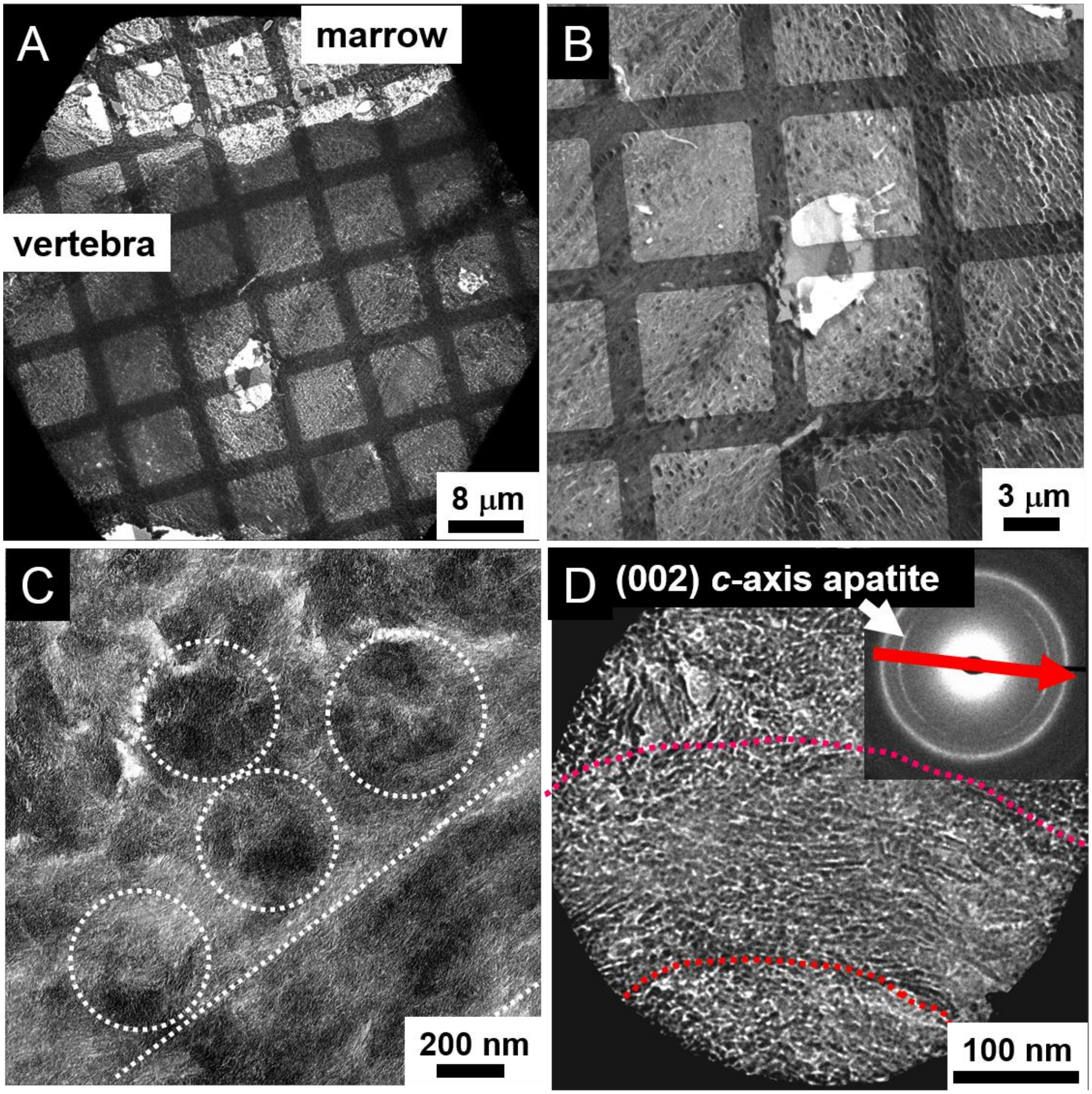

Figure S8: Healthy rat bone, ultramicrotomy, TEM images. (a) Overview of cross section of vertebra with bone marrow on top. (b) Zoom of central part. (c) High magnification image showing a fibril from the side indicated by dotted parallel lines and fibrils with long axis parallel to view direction marked by dotted circles. (d) Electron diffraction (inset right top) from a bend fibril indicated by white dotted lines. The diffraction pattern displays the preferred orientation of the apatite *c*–axis along the fibril long axis, which is typical for bone.

11. Figure S9

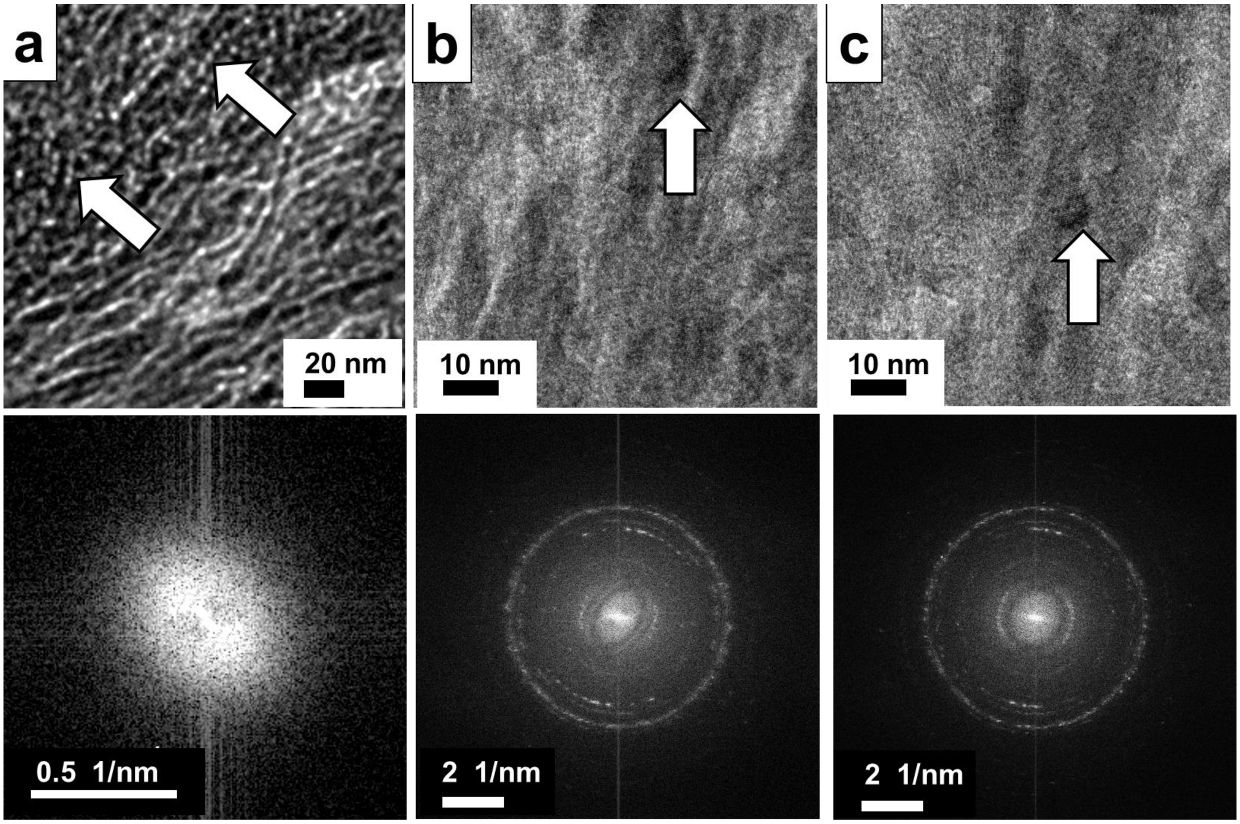

M =	32.000x	M =	320.600x	M =	320.600x
Drate =	5.4 e /Å^2	Drate =	900 e /Å^2 s	Drate =	850 e /Å^2 s
Dacc. ~	20 e/ Å^2	Dacc. ~	4500 e/ Å^2	Dacc. ~	178.500 e/ Å^2
Resolution:	15 Å	Resolution:	1.72 Å	Resolution:	1.72 Å

Figure S9. Overview and high–resolution TEM (top) together with FFT (bottom). The corresponding magnifications, dose rates and accumulated doses are noted below the images. (a) Overview image of Figure 1. At this magnification, the osteocalcin can be already observed at very low dose rate of about 5 e/sÅ^2. (b) High–resolution (top) image used for Figures 1,2,4,5,6. The dose rate amounts about 900 e/Å^2 and achieved resolution is 1.72 Å as read out from the FFT. There is no indication of occurrence of nanobubbles or for CaO formation. (c) High–resolution of slightly shifted area shown in (a). Defocus and astigmatism were adjusted with respect to (b). The arrow marks the same crystal in (b) and (c). Again, even at the accumulated dose of ~178.500 e/Å^2 the neighbor area is still well preserved.

12. Figure S10

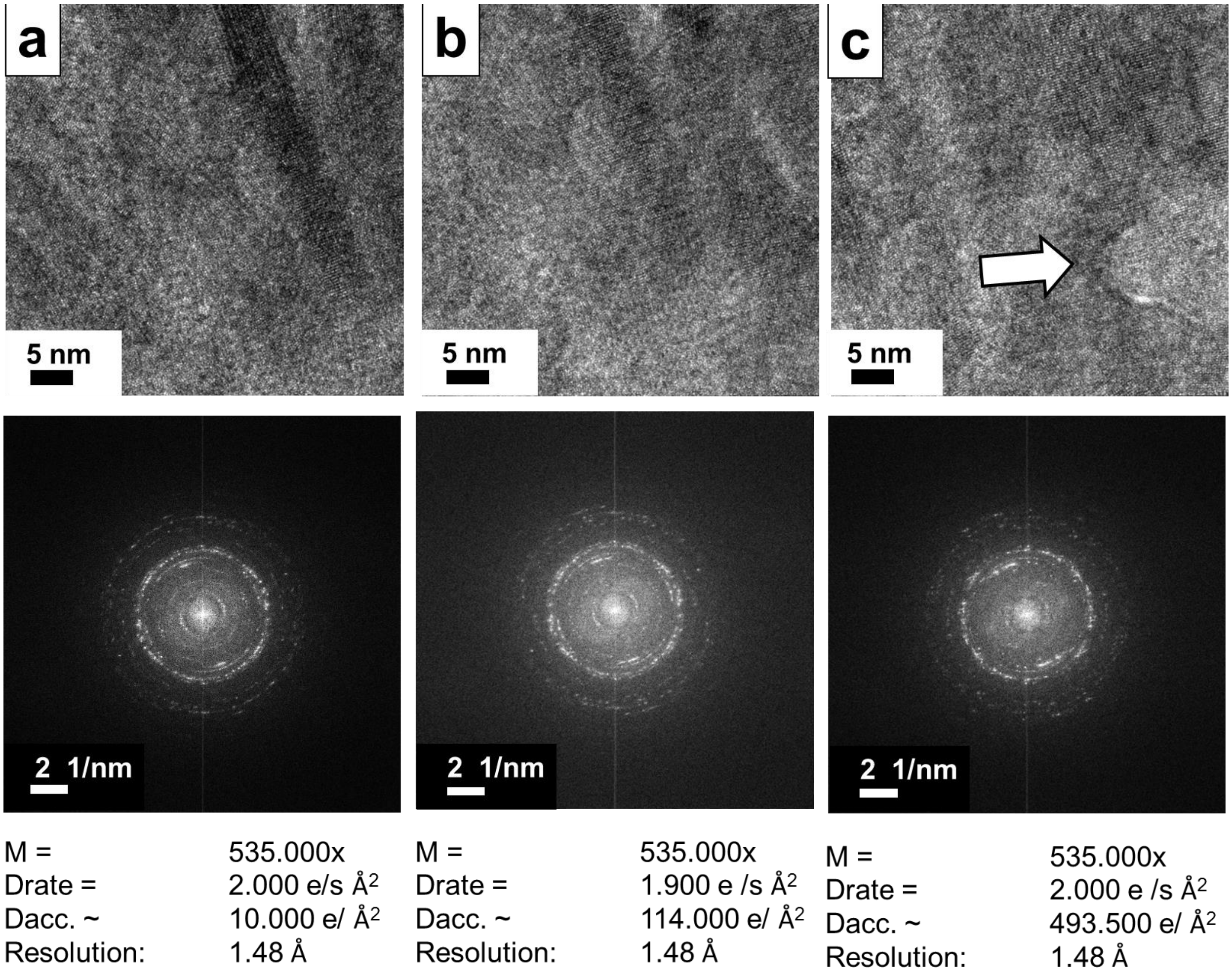

Figure S10. Time series beam damage experiments of FIB cut sample. High–resolution (top) and FFT (bottom) with corresponding magnifications, dose rates and accumulated doses. Magnification and dose rates were remarkably raised compared to the high–resolution image in Figure S9b which was used for final evaluation in Figures 1,2,4,5,6. (a) Starting image slightly defocused in order to achieve contrast. (b) After 1 minute accumulated dose reaches ~114.000 e/ Å^2. Structural changes in the direct image could not be observed and also the FFT shows no change indicating the same resolution as for the starting situation. After 3 minutes recording time at an accumulated dose of 493.500 e/ Å^2 formation of nanovoids occur (arrow).

15. Figure S11

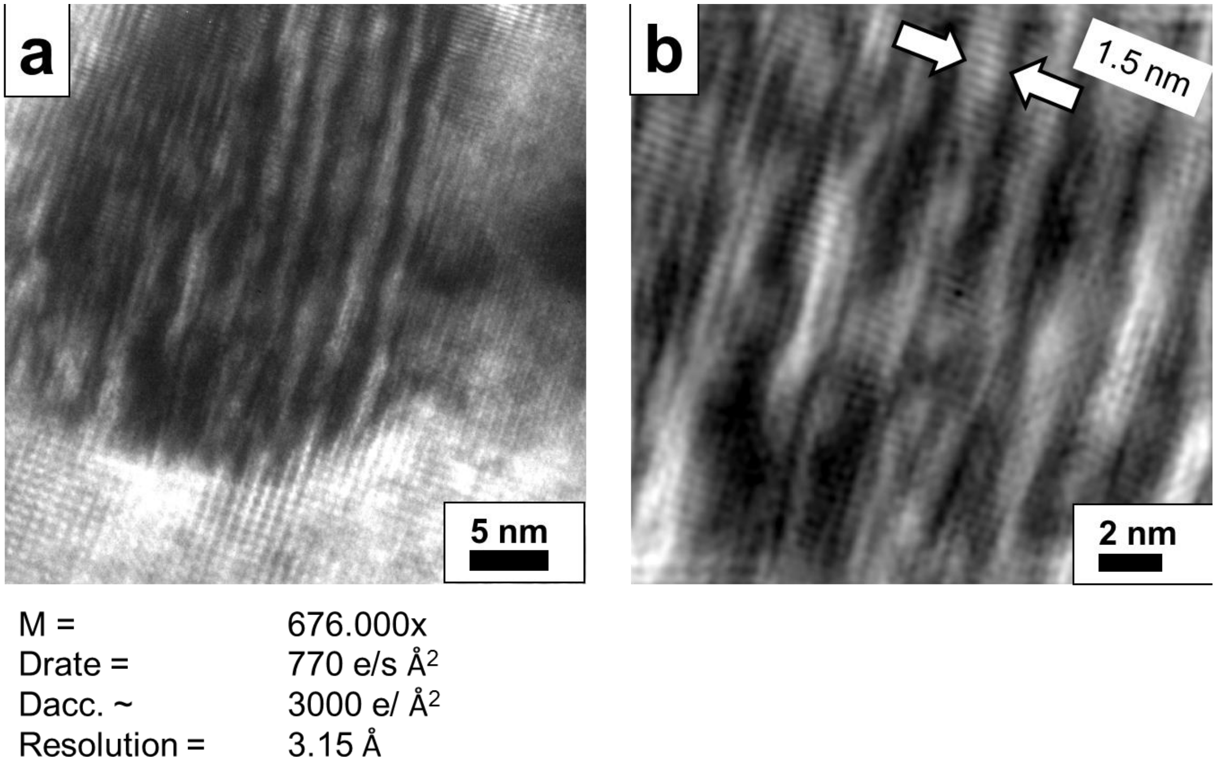

Figure S11. High–resolution TEM of FIB cut sample of fluorapatite–gelatine. (a) Triple–helical bundle embedded in apatite crystal. (b) Digitally zoomed area from the center of (a). The triple–helices are clearly imaged with the expected diameter of about 1.5 nm. Within the triple–helix the alpha–chain winding is revealed. On the left, a kind of disintegration of the triple–helical molecule is observed where the individual alpha–chains tend to a parallel arrangement, which may causes less mechanical stress with respect to the apatite lattice.[31]
